# Expected health risk out of black carbon and particulate matter in the indoor environment of an industrial cluster of chandigarh in India

**DOI:** 10.1038/s41598-025-01606-x

**Published:** 2025-07-02

**Authors:** Indramani Dhada, Sadiq Abdullahi Waziri, Vishal, Sudhakar Singha, Bijaya Kumar Padhi, Shailesh Kumar Samal

**Affiliations:** 1 Department of Civil Engineering, Indian Institute of Technology Ropar, Punjab 140001 Rupnagar, India; 2 Department of Civil Engineering, GITAM (Deemed to Be University), Hyderabad, 502329 Telangana India; 3 Community Medicine and School of Public Health, Post Graduate Institute of Medical Education and Research, Chandigarh, 160012 India; 4 Unit of Immunology and Chronic Disease, Institute of Environmental Medicine, Karolinska Institutet, 17177 Stockholm, Sweden

**Keywords:** BC, Lung function test, Lifetime cancer risk, Montecarlo simulation, Sensitivity analysis, Chandigarh, Environmental sciences, Environmental social sciences, Health occupations, Risk factors, Engineering

## Abstract

The global increase in industrialization and its attendant exponential air pollution has posed a significant hazard to the indoor pollution levels of cities and the associated health risks. This study evaluated the health effects of air pollutants discovered inside the bottling industries in Chandigarh cluster in India. PM_10,_ PM_2.5_, PM_1_, and black carbon concentrations in the post-monsoon season were monitored, and associated health implications and lung disease were estimated. A positive correlation is established between PM in indoor and outdoor environments. Maximum concentrations for PM_10_, PM_2.5_, and PM_1_ were recorded as 276.8 µg/m^3^, 97.7 µg/m^3^ and 66.5 µg/m^3^ (for indoor) respectively, which are approximately 15 and 6 times higher than their (PM_10_ and PM_2.5_) allowable concentrations set by World Health Organization, posing a health threat to the workers and staff of the industries. The lifetime carcinogenic risk of black carbon and the non-carcinogenic risk of particulate matter and black carbon have been assessed using a deterministic and probabilistic model, which shows the marginal difference. The estimated lifetime carcinogenic risk due to black carbon for males and females was observed in the range of 7.20E-05 to 6.17E-05. The spirometry analysis indicates that about 13.04% of the sample population (out of 184 samples) have healthy lungs.

## Introduction

In recent years, increasing air pollution has become one of the largest known environmental health threats. Studies have shown that exposure to air pollution is associated with both respiratory mortality and morbidity^1,2^. It is estimated that 91% of the world’s population lives in locations where air pollution (gases and solid particles) exceeded the permissible standard limits in 2016^3^. The combined effects of ambient air and household air pollution claim 6.7 million premature deaths worldwide annually. However, household air pollution alone is responsible for an estimated 3.2 million deaths per year in 2020^4^. Since people spend most of their time (around 80%) in a closed environment like an office, home, institution, industry, or other indoor areas^5,6^, and observe an increased concentration of air pollutants indoors than outdoors is attracting the concern of researchers to study various indoor micro-environments^7^. Some common indoor air pollutants reported by the literature survey are particulate matter (PM), volatile organic compounds (VOCs), black carbon(BC), and gaseous pollutants^8^. Exposure to PM produced by different manners of natural and anthropogenic activities is associated with various serious health effects, especially cardiovascular disease, pulmonary disorder, and cancer^9,10^.

PM is a mixture of different particles and liquid droplets, consisting of multiple components (e.g., organics, acids, metals, crustal material) and various size fractions^11^. Among the parameters that have a significant role in eliciting health effects are the surface and particle size, composition, and number, as they can absorb and transfer a multitude of pollutants^12,13^. Smaller particles such as BC, a fraction of PM_2.5_ emitted from the burning of fossil fuel^14^, and ultrafine particles (UFP; PM ≤ 100 nm)^15^ are of recent health interest. They can penetrate up to the lung alveoli due to their tiny size^12^ and are harmful through mechanisms of oxidative stress, cell signalling, activation, and release of mediators initiating inflammatory processes in the cardiovascular system and respiratory tract^9,12,16^.

BC is a component of aerosol that is produced by the incomplete combustion of biomass and fossil fuels^17^. It is a short-lived air pollutant with an average atmospheric lifetime of 4–12 days and a global warming potential of 460–1,500 times CO2^18^. It has detrimental effects on human health^19^ and the potential to pollute the indoor atmosphere in urban areas^17^, cryosphere (snow and ice) and agriculture^18^. Outdoor air pollutants have a significant contribution to indoor air pollutants^20^, and continuous exposure to those can be attributed to sick building syndrome (SBS)^21^. Although few studies were available on BC in indoor environments, it cannot be ignored as it contributes to respiratory diseases^22^. Toxicological research has indicated that BC can induce systemic inflammation and cause adverse damage to the lungs^23^, genotoxicity^24^, and has an effect on pulmonary function, and can cause myocardial dystrophy^25^. Epidemiological research shows that the concentration of atmospheric BC is linked to the morbidity of cardiovascular diseases^22^ and respiratory diseases^23^. Due to its Nano size, irregular morphology, and large surface area, it also acts as a receptor for PAHs and VOCs, which are carcinogenic and can pass through the bronchial tree and may cause serious health effects because they penetrate the human respiratory system^26^.

Lung function is an important measure of respiratory health and a predictor of cardiorespiratory morbidity and mortality^27^. Spirometry is a well-established tool of pulmonary ventilation tests to assess lung function and diagnose various pulmonary disorders in terms of lung capacity (LC), forced vital capacity (FVC), and forced expiratory volume in one second (FEV_1_)^28^. These parameters are compared with the reference parameters estimated with some standard empirical equations (ESCS), which consider age, weight, and height into account. A low FVC value corresponds to restrictive disease, while the decrement in FEV_1_/FVC is dedicated of an obstructive disease characterized by a kink in the spirogram^29^. Epidemiological studies suggest a negative correlation between PM concentration and lung function; similarly, acute exposure to automobile exhaust is associated with increased respiratory symptoms and may decrease and impair lung function^30^. Industrial pollution also poses a pulmonary threat, and several studies have suggested impacts from industrial air pollution, with strong support for effects on the development of lung function in people working in the industries and those residing in the proximate area^31^.

Further, the South Asian region has recorded one of the worst air pollution levels globally. In India alone, six hundred thousand premature deaths occur annually because of outdoor air pollution^32^. Approx. 80% of major cities in India exceeded the concentration of permissible limits for PM_2.5_ and PM_10_^33^. Although there is a plethora of literature available on air pollution and respiratory diseases, there is a paucity of studies on air pollution in an industrial cluster and the respiratory health of industrial workers exposed to the same environment. Also, there are fewer studies on industrial PM and BC aerosol concentration in the northern part of India. It is crucial to investigate BC and PM levels in the indoor environments of industrial clusters due to their severe health impacts. Industrial activities generate pollutants, which are known to exacerbate respiratory and cardiovascular diseases^34^. Moreover, the indoor environment is often poorly ventilated, and workers and residents spend extended periods indoors. Therefore, this study monitored concentrations of those pollutants along with estimating their health repercussion (using deterministic and probabilistic methods) inside the six bottling industries of UT Chandigarh City, which has a cluster of industries (> 2900 numbers). The air quality was monitored through portable instruments placed at inhalable height inside the bottling plants and just outside the gate of the bottling plant during working hours. In terms of health risk, carcinogenic and non-carcinogenic effects are estimated for BC and PM, respectively, while spirometry is used to assess lung health conditions. Some standard precision instruments like aerosol spectrometers and black carbon analyzers are used for pollutant concentration measurements over an 8-h working period, and an electronic spirometer is used for lung function tests. It will help to develop the knowledge over-concentration of PM and BC in the bottling industries of Chandigarh. Most of the previous research emphasize correlating the pulmonary disorder with ambient PM or BC concentrations, avoiding some other responsible factors, so the current study tries to incorporate all relevant sensitive factors using a questionnaire. This study carries great significance for public health, policy, and occupational safety. The findings can help develop indoor air quality standards, especially for industrial environments, where existing regulations are insufficient. Furthermore, the research can make policymakers and industry leaders support firmer regulatory frameworks to mitigate air pollution risks.

## Materials and methods

### Study area

**Fig. 1:**
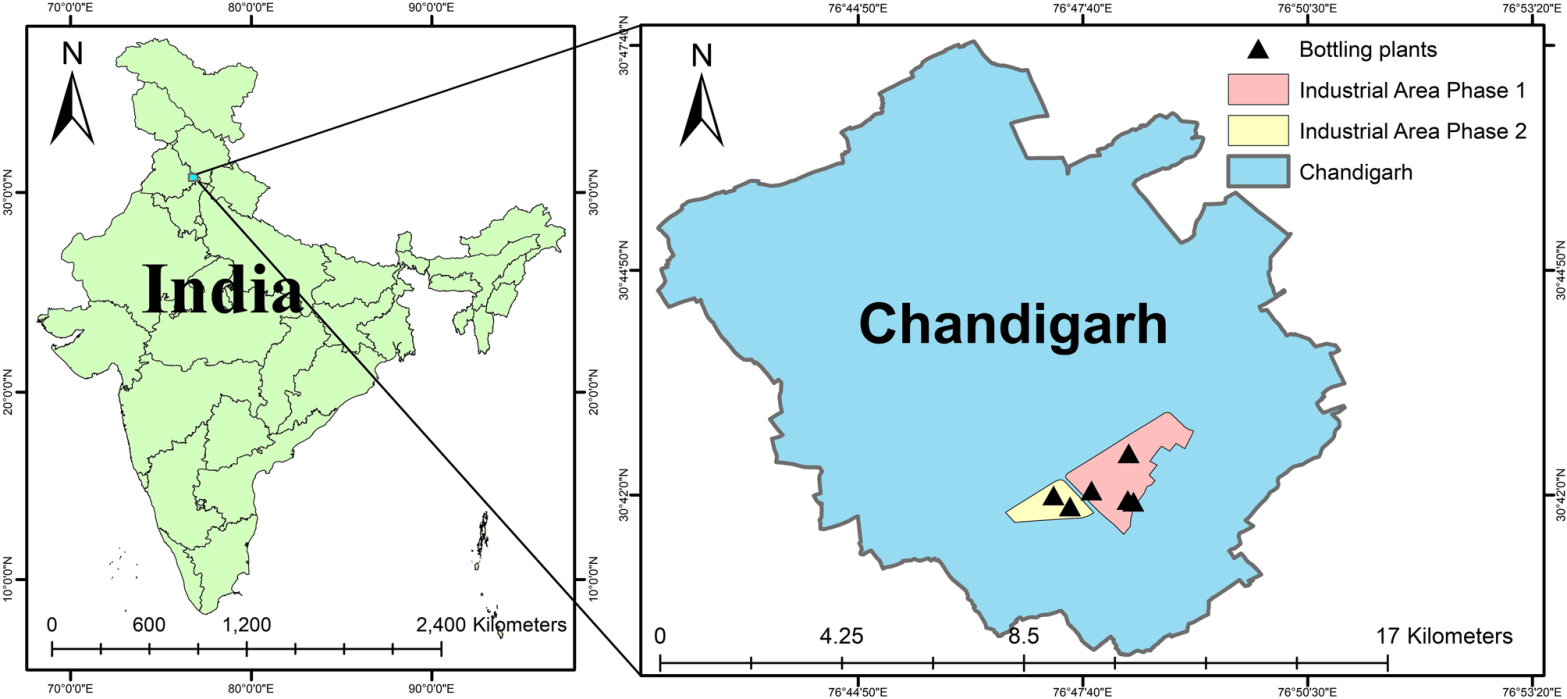
Schematic diagram of sampling location in UT Chandigarh.

The study was conducted in six bottling industries located in phases I and phase II of the industrial area (Fig. 1) in Union Territory Chandigarh (one of the developed and urbanized Indian cities (30.7333°N; 76.7794°E) to assess BC and PM concentrations during the post-monsoon season for 10 days (Late September and early October) of the year 2022. Chandigarh has a humid subtropical climate with great temperature variation (− 1 to 45 °C) annually, but had a mean temperature and RH, 35 °C and 47% (outdoor) and 29 °C and 70% (indoor) during the monitoring period. Chandigarh is an industrial hub having good industrial density, and there are about 2950 small-scale and 15 large and medium-scale units existing in Chandigarh^35^.

Targeted industries were adjacent to main roads and mainly faced vehicular pollution. All industries lie within a radial distance of 1 km. Approximately 40–50 employees were working in each industry, either as workers or staff, with age variation between 18 to 60 years, comprising both males and females in the working hours of the industries (9 AM – 5 PM IST). The industrial operations being carried out in industries were rinsing and cleaning, blending, filling, capping and sealing, labelling, packaging, and dispatching. It was noticed that there was improper ventilation in the indoor environment.

### Air quality data and sampling methodology

The pollutants measured consisted of fine PM ranging from 0.25 µm to 32 µm (in 31 channels) and BC in industries’ indoor and outdoor environments. The instruments were kept in the area as close as possible to the breathing zone (around 1.5 m height) during the study period (9 AM – 5 PM IST) so that the actual inhalable concentration could be recorded while avoiding any kind of obstruction near the sampler’s inlet nozzle to a 3-m distance. The data collection was done during the same working hours of 9 AM – 5 PM IST within the industries.

Air quality data in terms of PM and BC in indoor as well as outdoor environments was assessed with the help of a portable battery-operated laser photometer (Aerosol spectrometer, model 1.109, make: GRIMM, USA) and optical sensor-based handy instrument (Black Carbon Analyzer, make: Distributed Sensing Technologies, California) respectively. Along with this, ambient concentrations of PM_2.5_ and PM_10_ were also taken from a nearby fixed monitoring station owned by CPCB (Central Pollution Control Board) located at sector 22 (around 4 km aerial distance from the plant site) for the same sampling duration and the annual period from the CPCB website to get a comparison between the two outcomes. Then, an annual indoor concentration was proposed for PM_2.5_ and PM_10_ using Eq. 1.

1 $${C}_{4}=\frac{{C}_{1}{C}_{2}}{{C}_{3}}$$

where, C_1_: average ambient concentration of CPCB data for one year.

C_2_: 8-hour average concentration of pollutants in the indoor environment of industries over a study duration of 10 days (recorded by sampling instrument)

C_3_: average concentration of pollutants in ambient air, recorded by CPCB (for 10-day study duration).

C_4_: Proposed 8-h annual average indoor concentration in industries.

### Estimation of health risk

All sampling methods were carried out following relevant guidelines and regulations.

and all experimental protocols were set, incorporating prior ethical informed consent from industrial owners. The health risk has been estimated considering the annual average indoor pollutant concentration (proposed C4: as estimated from Eq. 1) in terms of hazard index (HI) for PM and BC as non-carcinogenic risk and lifetime cancer risk (LCR) for BC following the deterministic as well as probabilistic approach of estimation. Since physiology and behaviour vary according to age, weight, and sex^36^, the population is divided into five age categories; 16–21, 21–30, 30–40, 40–50, and 50–60 years for male and females individually and the average values of parameters for each category is taken from the exposure handbook of USEPA^37^. Then, the health risk is estimated for all age groups by taking the average indoor concentration (over 10 days) and proposed annual average indoor concentrations of pollutants obtained after using a suitable conversion factor as described in Eq. 1. The two approaches used for risk calculation are as follows.

#### Deterministic health risk

The risks are estimated based on the health risk assessment model of the USEPA 2011^38^. Since the employees of industries belonged to different age groups, health risk was estimated considering the variability of parameters (Table 1). Similar guidelines were also followed by Deng et al. for PM_2.5_^39^, Li et al. for PAHs^40^, and Bakhtiari et al. for H_2_S^41^ gas in health risk estimation. The daily intake of pollutants (Eq. 2), their reference dose (Eq. 3), and associated hazard quotient (Eq. 4) have been assessed.

2 $$CDI=\frac{CA\times IR\times ET\times EF\times ED}{BW\times AT}$$

where CDI is chronic daily intake (mg/kg/day), CA is the average concentration of pollutant (mg/m^3^), IR is inhalation rate (m^3^/hour), ET is the exposure time (hours/day), EF is exposure frequency (days/year), ED is exposure duration (years), BW is mean body weight (Kg) and AT is averaging time in days (taken equal to ED for non-carcinogenic and 70 years for carcinogenic risk). For the current study, IR and BW correspond to the average values suggested by the manual, while CA, ET, EF, and ED are used particularly as per our primary data collected at the site. These values are comprised in Table 1: Mean values of parameters considered in health risk estimation, suggested by USEPA (2011) using exposure time, frequency and duration from field data.

**Table 1:** Mean values of parameters considered in health risk estimation, suggested by USEPA (2011) and on-spot survey.

	Males					Females				
Age (years)	16–21*	21–30	30–40	40–50	50–60	16–21	21–30	30–40	40–50	50–60
Mean Inhalation Rate (m^3^/day)	17.21	18.82	20.29	20.94	20.91	13.59	14.57	14.98	16.2	16.19
Mean Body Weight (kg)	77.3	84.9	87	90.5	89.5	65.9	71.9	74.8	77.1	77.5
Exposure Time (hours/day)	8	8	8	8	8	8	8	8	8	8
Exposure Frequency (days/year)	313	313	313	313	313	313	313	313	313	313
Exposure Duration (years)	35	35	35	35	35	35	35	35	35	35
Averaging Time (years)	70	70	70	70	70	70	70	70	70	70

*The age range is one year less than the upper limit e.g. 16–21 can be read as 16 to 20 years, 21–30 would be 21 to 29 years and so on.

3 $$RfD=RfC\times IR/BW$$

Here, RfD (mg/kg/day) is the reference dose, and RfC is the permissible concentration of the pollutant. This study considers the permissible concentrations suggested by the CPCB (Central Pollution Control Board) and WHO (World Health Organization), which are given in Table S1. Due to the absence of permissible concentration over an 8-h average, this study incorporates the RfC of 24-h average concentrations prescribed by CPCB and WHO.

Further, formulae considered for the estimation of hazard quotient (HQ) and HI are given in the following equations:

4 $$HQ=\frac{CDI}{RfD}$$

5 $$HI=\sum HQ$$

Black carbon is hazardous due to its characteristics and nano-sized particles and is also recognized as carcinogenic if it is emitted from diesel soots^42^. Carcinogenic risk due to BC is estimated in terms of LCR using Eq. 6. Slope factor (SF), taken as 1.1 mg/kg-day by considering the risk of BC, equivalent to risk due to diesel engines exhaust^43,44^.

6 $$\text{LCR}=\text{CDI}\times \text{SF}$$

The limits for HI and LCR beyond which the pollutants may cause significant non-carcinogenic and carcinogenic risks are 1 and 1 × 10^–6^ respectively^45^. The EPA considers the carcinogenic risk to be negligible if there is less than one case of cancer in a population of one million persons (1 × 10^–6^) and if the risk is greater than one chance in ten thousand persons (1 × 10^–4^), the risk is sufficiently high and medical attention is required.

#### Probabilistic health risk

This study follows both the deterministic as well as probabilistic approaches because the estimation of risk with a deterministic model may probably contain some errors or unrealistic results due to the consideration of mean values instead of their real varying values for independent risk variables under consideration^46,47^. In this context, a probabilistic approach may be a more realistic way to estimate health risk, which can eliminate the impact of uncertainty involved due to varying environmental conditions and individual health characteristics^48,49^. Therefore, in the present study, a Monte Carlo (Crystal Ball 11.1.2.4.850; ORACLE) simulation-based probabilistic approach was adopted for the computation of health risk along with a deterministic approach. A total of 50,000 iterations were performed with the risk variables. The probability distributions of all the adopted risk variables are given in Table 2. Moreover, parameter sensitivity analysis was also performed to determine the importance of each health risk variable in HR computation.

**Table 2:** Probability distributions of all the adopted risk variables.

Parameter	Symbol	Units	Distribution	Reference
Concentration of pollutant	CA	mg/m^3^	Log-normal	Current study
Inhalation rate	IR	m^3^/hour	Normal	(Giri et al., 2020)
Exposure Time	ET	hours/day	Uniform	Current study
Exposure frequency	EF	days/year	Triangular	(Smith, 1994)
Exposure duration	ED	years	Uniform	USEPA 1991c; 2011
Average body weight	BW	kg	Normal	ICMR 2010
Reference doses	RfD	mg/kg/day	Point	(USEPA, 1989)

### Pulmonary function test

Pulmonary function tests (PFT) were carried out following the guidelines of the American Thoracic Society (ATS 1995) using a portable, electronic spirometer (Contec SP-10BT) with a disposable mouthpiece, designed for pulmonary function measurements. The device measures actual expiratory flow at a precision of 2%, in addition to predicted values according to age, sex, height, weight, and smoking habit with available empirical equations, i.e. ECSC^50^. Before performing the PFT, the procedure was well demonstrated to the respondents, and the height and weight of the respondents were measured with shoes removed. Employees (staff members and workers), after informed consent has been taken, performed forced expiratory manoeuvers while sitting with free mobility and nose closed with a nose clip to prevent the passage of air through the nose. Respondents who were facing any kind of illness were excluded from the sample population^51^. Test samples were taken in triplicate, considering the highest values to avoid error, and any two values of FEV_1_ should not differ by more than 5% according to the ATS criteria. Using a computer-assisted quantitative assessment, the best manoeuvre for acceptance was determined.

A flow plotted against volume to display a continuous loop of expiration was extracted, as the shape of the flow volume loop is important for interpreting spirometry indices and results. The specific spirometry parameters (absolute and relative values and the ratio of actual and predicted values) such as forced vital capacity (FVC), forced expiratory volume at 1 s (FEV_1_), the ratio of FEV_1_ to FVC (FEV_1_/FVC), expressed as a percentage and peak expiratory flow rate (PEFR) during expiration were recorded for analysis. Decrement of lung function detected by spirometry could be generally of two types: obstructive type and restrictive type of impairment. In some cases, combined (both obstructive and restrictive) types of lung function impairment could also be encountered.

### Pulmonary disorder estimation

**Fig. 2:**
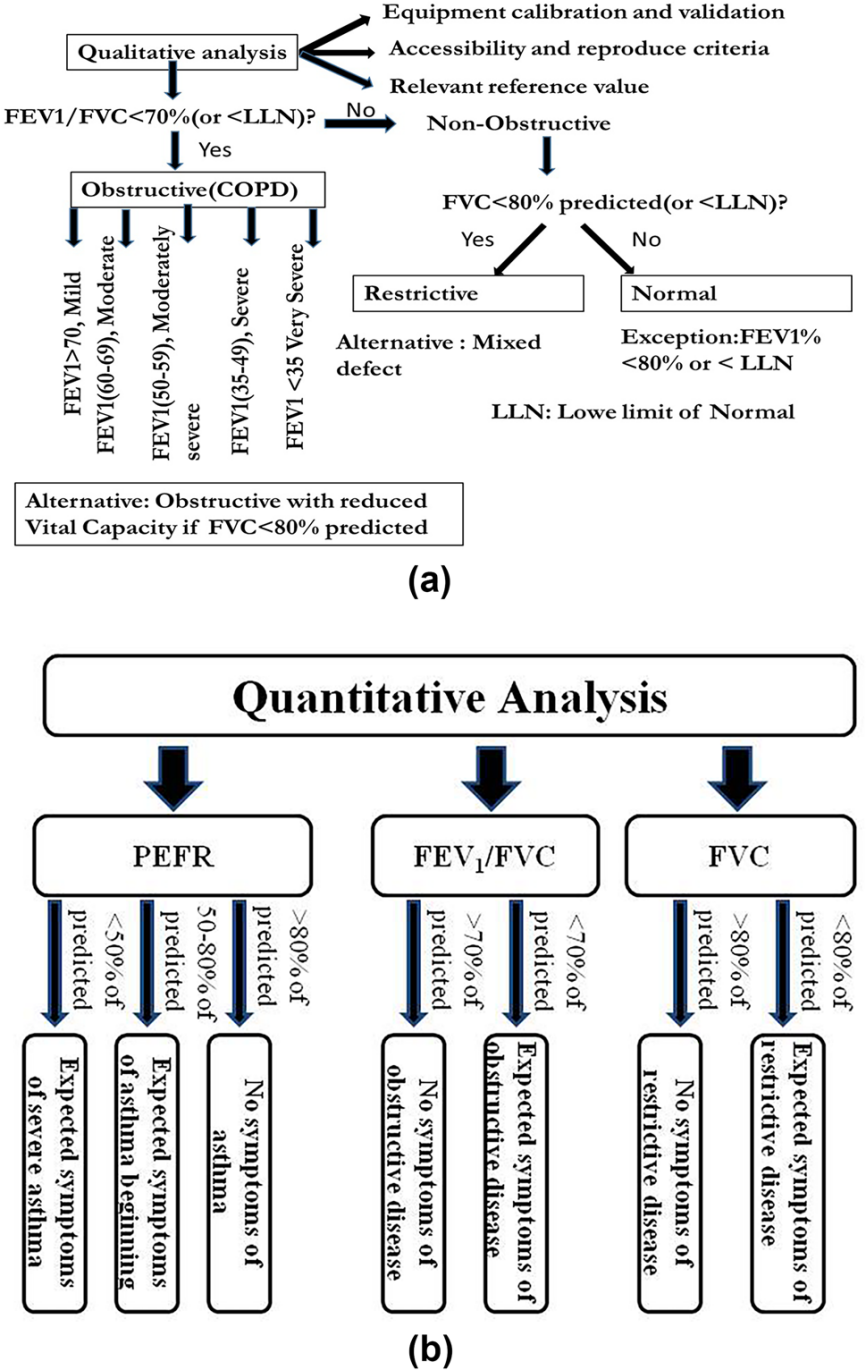
(**a**) Algorithm for pulmonary disease assessment (qualitative analysis) (**b**) Algorithm for pulmonary disease assessment(quantitative analysis).

Three common pulmonary disorders are considered in this study, i.e. restrictive lung disease, obstructive lung disease, and asthma, which have been estimated using the algorithm given in Fig. 2^29,52,53^. In obstructive types of lung disorder, deficits such as emphysema or chronic bronchitis, the FEV_1_ is reduced disproportionately more than the FVC, resulting in an FEV_1_/FVC ratio of less than 70%. Thus, FEV_1_/FVC < 70% diagnoses airway obstruction, which has been considered in this study. Literature suggests that subjects with obstructive lung disorder have a rapid peak expiratory flow, but the curve descends more quickly than normal and takes on a concave shape, reflected by a marked decrease in the FEF25–75%. With more severe obstruction, the peak becomes sharper, and the expiratory flow rate drops precipitously^29^. Similarly, in restrictive lung impairment, the FVC is reduced below 80% of the predicted value. The shape of the flow volume loop is relatively unaffected in restrictive disease, but the overall size of the curve appears smaller when compared to normal on the same scale. Apart from this, one can also be suffering from two diseases simultaneously. Subjects having this problem have FVC less than 80% of the predicted value and FEV_1_/FVC ratio < 70%.

For assessing the prevalence of asthma, peak expiratory flow rate (PEFR) is considered as an indicator following the guidelines of NIH (National Institutes of Health through the National Asthma Education and Prevention Program)^54^. NIH classified asthmatic conditions into three categories based on PEFR value predicated on the percentage of observed PEFR value, which is: (a) no symptoms of asthma when observed PEFR value exceeds 80% of the predicted value; (b) mild symptoms of asthma, when observed PEFR value, lies between 50 and 80% of the predicted value (considered as the beginning of asthma); and (c) severe symptoms of asthma when observed PEFR value is less than 50% of the predicted value (requires medical attention). An individual’s predicted PEFR value (or standard) depends on sex, age, and height.

### Response of participants

Along with spirometry tests, a well-structured questionnaire (based on a literature review) was also floated for the sample population to assess the contribution of some pre-searched factors (caused by exposure to various environments) which can influence lung functioning. All the questions were clarified to respondents by giving additional explanations by the interviewers. The questionnaire constitutes “exposure time in the industry, consuming alcohol, smoking, type of cooking fuel at home, use of exhaust at home, the proximity of residence to the highway, dust allergy, height, weight, history of lung disease, etc. A total of 184 respondents were administered to staff members/workers after furnishing the ethical formalities. The information collected from the respondents were; name, age, gender, height, weight, daily exercise duration (hr) and frequency of exercise (days per week), smoking habit (active or passive), alcohol consumption, dust allergy symptom, clinically asthma symptom (of self and family members), a symptom of lung disease (self and family members), designation as worker/staff, industry working hr/day, a working period in that industry (years), home located in an urban or rural, home situated < 100 m or > 100 m from the roadside, the kitchen garden at home, use of exhaust fan in kitchen, ventilation in rooms, numbers of occupants at residence, Fuel use at home(wood/LPG) and its usage duration(hr), and use of insecticide or pesticide at home.

## Results and discussion

### Particulate matter (PM)

**Fig. 3:**
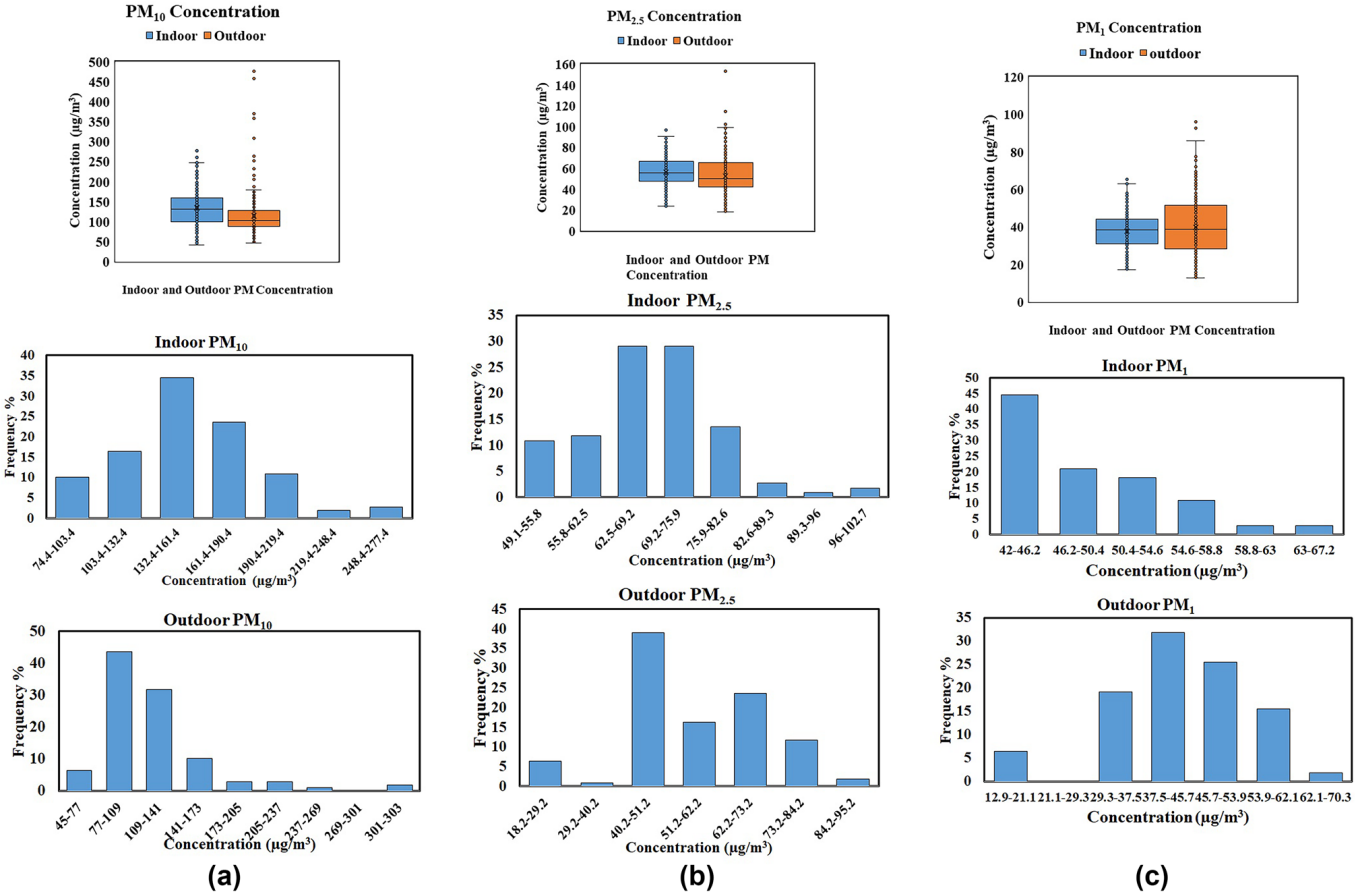
(**a**) Boxplot and Histogram for PM_10_ (**b**) Boxplot and Histogram for PM_2.5_ (**c**) Boxplot and Histogram for PM_1_.

This study monitored the mean concentrations of PM for indoor as well as outdoor (just outside the entrance) environments of six bottling industries over 10 10-day period in the post-monsoon (Late Sept–Early Oct) season, situated in an industrial area of Chandigarh City. The mean temperature and relative humidity recorded by CPCB Chandigarh city during the sampling period were found to be 32.21 ± 2.69 °C and 47 ± 3.23, with northern winds most of the time. Since very few variations in these meteorological parameters were observed during the sampling period, the effect of these parameters could be neglected in pollutant dispersion. The 8-h average concentrations of PM_10_, PM_2.5,_ and PM_1_ were recorded to be 115.86 ± 21.87 µg/m^3^, 52.12 ± 4.98 µg/m^3^, and 39.28 ± 5.90 µg/m^3^ just outside the entrance of the industries, while in the indoor environment, these concentrations were 131.29 ± 12.35 µg/m^3^, 54.51 ± 1.91 µg/m^3^ and 35.83 ± 3.27 µg/m^3^, respectively. Mean concentrations of PM_10_ and PM_2.5_ indoors were higher than outdoors by about 13% and 5%, while the concentration of PM_1_ was higher outdoors than indoors by nearly 10%. The reported concentrations were the mean over an 8-h sampling period and can be compared with standard limits. Due to the unavailability of permissible limits/standards over an 8-h average for the indoor environment, the monitored concentrations of PM_10_ and PM_2.5_ were compared with permissible limits suggested over a 24-h daily average period set by the national ambient air quality standard of the central pollution control board (NAAQS-CPCB) (PM_10_ ≤ 100 µg/m^3^, PM_2.5_ ≤ 60 µg/m^3^; NAAQS^55^; and WHO (PM_10_ ≤ 45 µg/m^3^, PM_2.5_ ≤ 15 µg/m^3^^56^;, similar to other studies i.e^39,57–59^.. It was noticed that mean concentrations of PM_10_ were higher than their allowable limits (NAAQS as well as WHO) inside as well as outside the industries. However, mean PM_2.5_ readings were approaching NAAQS standards and were much more than the permissible limit of the WHO. Mean concentrations of PM_10_ and PM_2.5_ were higher than the allowable concentrations set by WHO by 2.6 and 2.2 times indoors and 2.3 and 2.1 times outdoors, respectively. PM_1_ encompasses a very tiny size, is capable of easily penetrating the alveolar region of the lungs, and hence is more fatal than PM_2.5_ and PM_10,_ and its presence is objectionable to the environment even with low concentration^60^. Since there is no permissible limit defined for PM_1_ yet by any regulatory body, but poison effect of the observed concentration can not be underestimated. A study conducted in two hospitals in China revealed a rise in hospital admissions associated with a higher concentration of PM_1_^61^. However, the mean concentrations of PM_10_ and PM_2.5_ were higher indoors, but Fig. 3(a) and Fig. 3(b) showed a lower mean in the outdoor environment with many outliers than indoors, which explained the sudden fluctuation in PM levels outside the industries. Since the target industries were adjacent to the road and surrounded by a cluster of small-scale industries, outliers could be attributed to high traffic volume or other industries during working hours in the daytime. Studies also suggest that the PM variation depends on traffic density^62^.

While correlating the concentrations of PM_10_ with PM_2.5_, a strong positive correlation was observed (Appendix A) with r = 0.77, p-value < 0.001 (for indoor) and r = 0.68, p-value < 0.001 (for outdoor), which ascertained common sources of pollutant i.e. automobile exhaust, road traffic dust, industrial emissions, and crowd intervention, etc. No such significant peaks or outliers for PM_10_ and PM_2.5_ concentrations are visible indoors, which indicates the absence of local sources in the indoor sampling area. Results also found that the air pollutants are similar, and the ratio of pollutant concentration found indoors to outdoors is > 1. This could be attributable to the penetration of pollutants from outside through the aperture of doors and windows, and was higher due to the compact working space, workers’ activities (which often result in the resuspension of dust particles) and lack of proper ventilation during industrial operations. Earlier studies suggest that ventilation also plays an important role in reducing pollutant concentrations^63^. Hence, increased PM levels could also trigger poor ventilation in target sites.

Further, the maximum concentrations observed for PM_10_ and PM_2.5_ in the industries were recorded as 276.8 µg/m^3^ and 97.7 µg/m^3^ (inside) and 736.3 µg/m^3^ and 153.1 µg/m^3^ (outside), respectively, which is approximately 2.7 times more outdoors than indoors (for PM_10_). Similarly, the maximum and minimum values in the indoor environment for PM_1_ were detected to be 66.5 µg/m^3^ and 17.1 µg/m^3^. However, the histogram (Fig. 3) showed that the most frequent concentrations lie in the range of 132.4–161.4 µg/m^3^, 62.5–75.9 µg/m^3,^ and 42–46.4 µg/m^3^ for PM_10_, PM_2.5_, and PM_1,_ respectively. While considering the mass concentration of PM_10_ and PM_2.5,_ it followed a normal distribution, while PM_1_ exhibited a lognormal distribution in the indoor environment. Also, the concentration of indoor PM_1_ lies in a low range most of the time and rarely shows increments in values. On the other hand, outdoor concentrations show a good occurrence of high concentrations. For PM_10_, the maximum concentration was higher (for a short time) outdoors, but most of the time, the concentration was found in the range of 77–109 µg/m^3^, compared to indoors (132.4–161.4 µg/m^3^). This indicates that more health threats are expected due to the PM inside the industries.

This study recorded concentrations (131.29 µg/m^3^) of indoor PM_10,_ which was similar to the reported value of 139.28 µg/m^3^ (at Khrew) by Qadr, a study conducted in April 2023 for nine cement industries situated in Khrew and Khanmoh, Kashmir Valley^64^. However, the PM concentrations in the casting industry of North India^65^ and stone-crushing industries^66^ showed copious increments (ranging in mg/m^3^) than the observed concentrations in this study. In some other indoor environments like roadside educational institutes, the concentrations reported were 61±29 µg/m^3^ for PM_2.5_ in Chennai^62^ and 157.80±67.84 µg/m^3^, 92.48±45.74 µg/m^3^ and 67.46±31.12 µg/m^3,^ respectively for PM_10_, PM_2.5_ and PM_1_ in Agra city of India^58^ and are higher than those observed in this study. The observed PM_2.5_ concentration found in the bottling industries of Chandigarh was in a similar range reported in a newly built residential building in Xi’an, China^67^. Also, the concentrations reported by Eghomwanre^59^ in Benin City of Nigeria, for PM_10_ (106.1µg/m^3^), PM_2.5_ (93µg/m^3^), and PM_1_ (49.4µg/m^3^) were in good agreement with the outdoor concentrations observed in the current study. However, compared to the WHO permissible limit (24-hour average: 45µg/m^3^ for PM_10_, 15µg/m^3^ for PM_2.5_), the targeted industries in the present study appear to be affected by higher concentrations of PM from outside.

### Black carbon (BC)

**Fig. 4:**
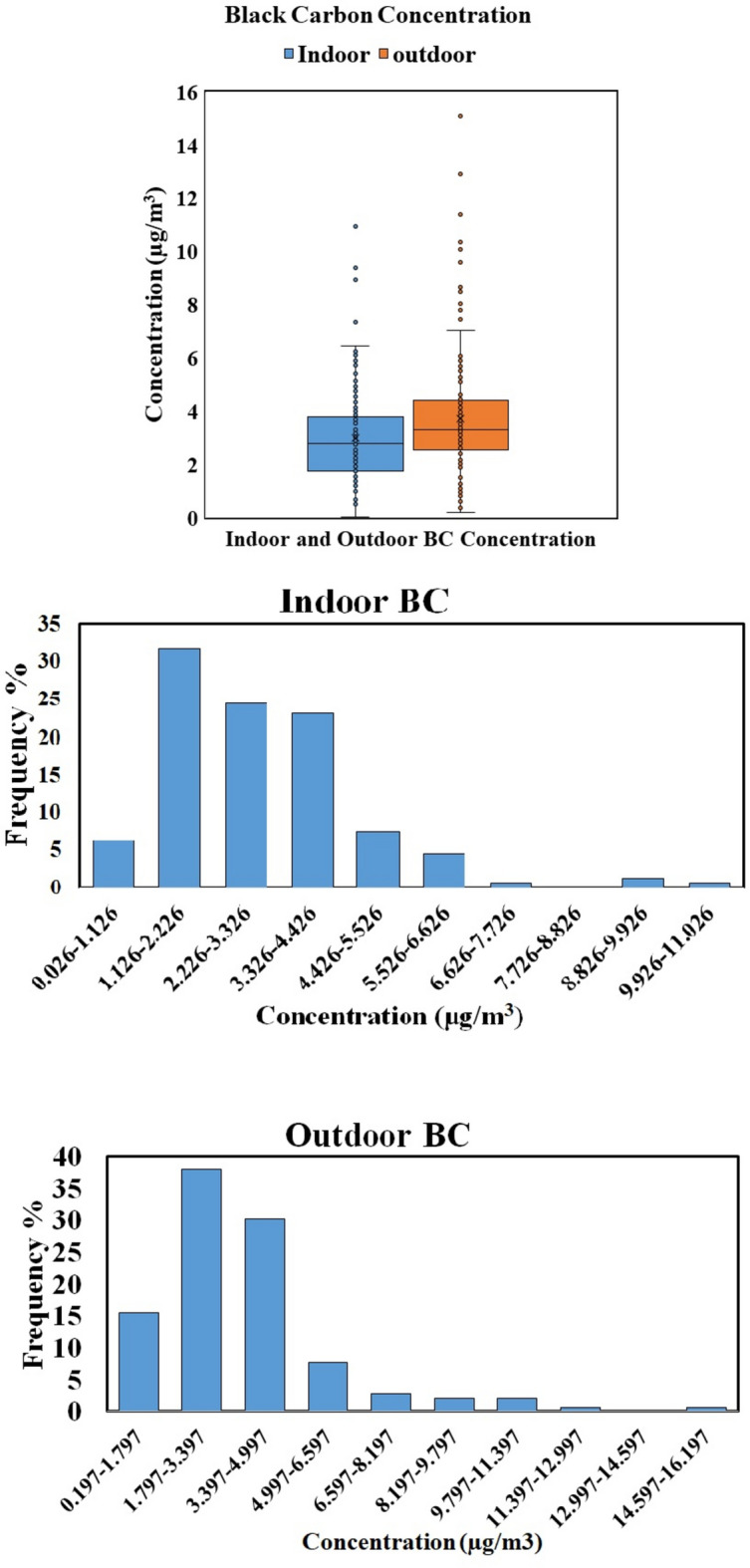
Boxplot and Histogram for BC.

The sampling was performed in bottling industries during working shifts for 8 hours, and the average of daily mean concentrations of 10 days of sampling (spread over two months) was taken for analysis. The mean concentrations of BC in the outdoor (just outside the entrance) and indoor environments were recorded as 3.76 ± 1.04 µg/m^3^ and 2.94 ± 0.93 µg/m^3,^ respectively. Fig. 4 shows a boxplot illustrating the average, minimum, and maximum concentration of BC found in those industries, where the outdoor concentrations had high peaks with the maximum concentration recorded as 15.07 µg/m^3^, but in the case of the indoor environment, it was 10.94 µg/m^3^. A positive correlation (r=0.52, p<0.01) between the outdoor and indoor concentration of BC exists, elucidating the significant contribution from outside pollution sources to the indoor area of industries. Also, the daily average PM_2.5_ and BC concentrations were highly correlated (r = 0.92) inside the industries.

The outdoor source of BC may be vehicular exhaust along with other industrial fuel burning in that cluster, which comprises approximately 2900 industries. The observed indoor source for BC concentration could be a silent diesel generator (DG) used for electricity generation during power fluctuations. To examine the most prevalent range of BC, a histogram analysis was carried out for the observed concentrations inside as well as outside of target industries and is shown in Fig. 4. The most frequent concentration range observed indoors was 1.12–2.22 µg/m^3^, contributing to 32% of the sampling period, while for outdoors it was in the range of 1.79–3.39 µg/m^3^ for nearly 38%. The BC concentration was not reported by CPCB for the study site. However, the 8-hour average permissible limit for BC set by NIOSH of OSHA is 3.5 mg/m^3^ (without the presence of PAHs), which is higher than the observed range recorded in this study. Furthermore, a study from North-Indian villages reported a mean concentration of 14.54 µg/m^3^ of BC found in rural household kitchens^68^, which is much higher than the values observed in this study. This is due to the burning of fossil fuel (coal and wood) in households, while no such fuel burning was found within the bottling industries in the present study, except for diesel generator sets, resulting in lower concentration.

Since the particle size of BC contributes to a major range of PM_**2.5**_, it shows health effects that are similar to PM_2.5_^69,70^. Reported literature reveals the effects of BC on respiratory, cardiovascular, and ocular diseases with a mean concentration of 5.2 µg/m^71^. It was also found that there is an association between emergency myocardial infarction hospitalization and the BC concentration exceeding 1.7 µg/m^72^. Similarly, an increase in PM_2.5_ and BC was associated with an increase in emergency room visits reported in hospitals in Shanghai^73^. In addition, there is evidence that combustion-related components of PM are more harmful than non-combustible fractions^74^.

### Correlation between PM and BC

**Fig. 5:**
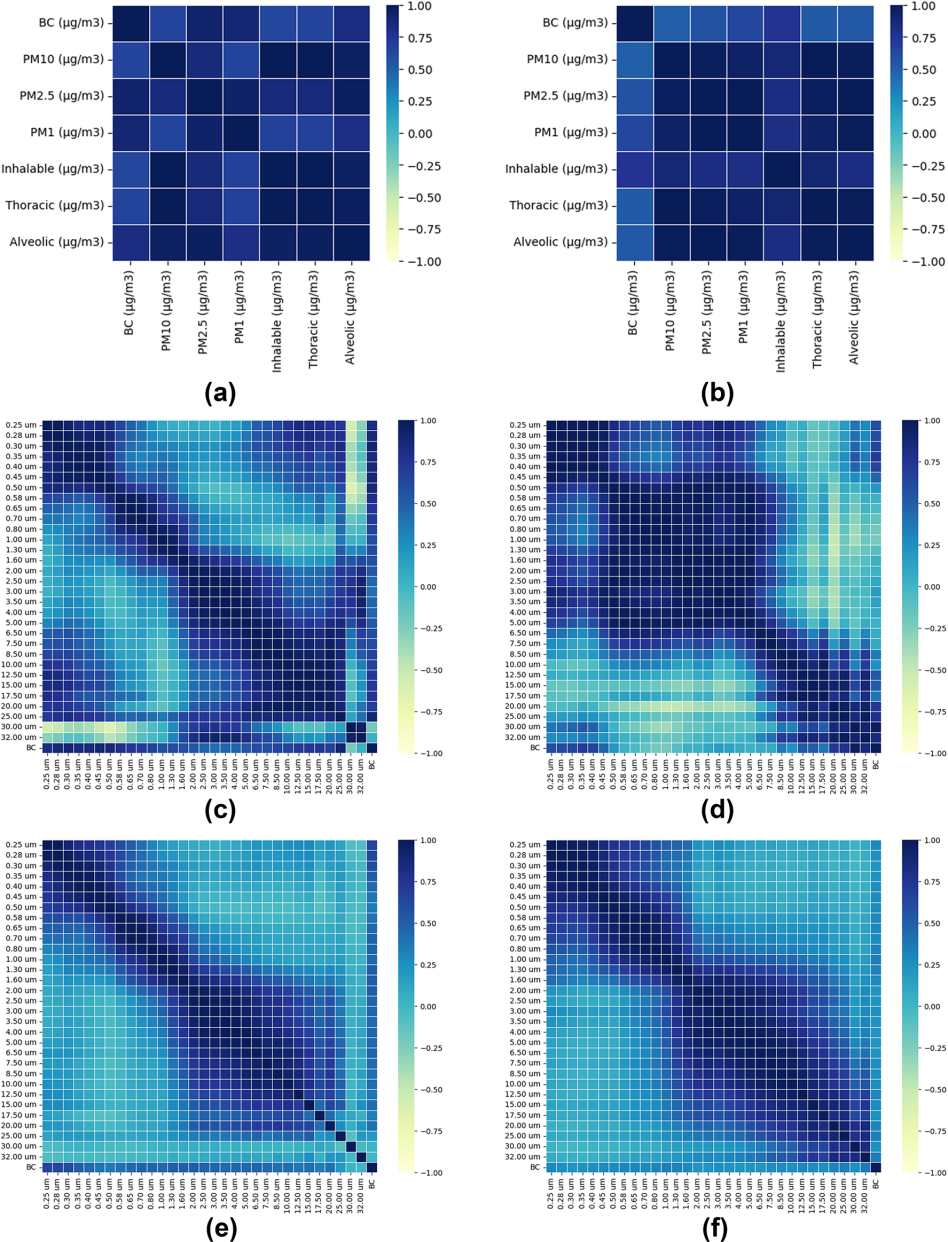
(**a**) Correlation between mass concentration of PM (3 ranges) and BC for indoor (**b**) Correlation between mass concentration of PM(3 ranges) and BC for outdoor (**c**) Correlation between mass concentration of indoor PM (31 ranges) and BC (**d**) Correlation between mass concentration of outdoor PM (31 ranges) and BC (**e**) Correlation between the number concentration of indoor PM (31 ranges) and BC (**f**) Correlation between the number concentration of indoor PM (31 ranges) and BC.

The Pearson correlations between mass concentration of various ranges of PM and BC are depicted in Fig. 5. A strong correlation of BC was found with indoors PM_1_ and PM_2.5_ (Fig. 5(a)), whereas, for outdoors, BC has a significant positive correlation with inhalable PM ranging from 10µm to 34 µm along mild correlation with PM_2.5_. This concludes that for the outdoors, the source of BC (10µm to 34 µm) is from other industrial activity(might be burning of fuel), along with vehicular pollution. Burning of fuel (coal, tires, wood, etc) releases a higher amount of unburnt carbon (in a higher range) than vehicular exhaust^75^. A cloud of visible smoke in case of fuel burning can be seen (showing a good concentration of soot particles), which is comparatively very less in vehicular exhaust due to pollution mitigation measures installed within the vehicle^76^.

The present study reveals that the major size range of indoor BC lies in PM_2.5_ and PM_1,_ similar to other reported ranges^77–79^. So, the primary sources of BC were vehicular exhaust and diesel generator exhaust, as it is highly correlated with PM_2.5_ and PM_1,_ which is similar to the results reported by Rarpathi and Phuleria^79^, who observed the BC concentration positively correlated with PM_2.5_ when the emission was from the vehicular exhaust. When BC concentration was compared with a 31-channel range of PM (Fig. 5(c), 5(d)), 5(e) and 5(f) varying from 0.25 µm to 32 µm, it was found that BC was significantly correlated with ranges 0.25–0.45 µm and 20–32 µm (for outdoor, Fig. 5(b)) and 0.25–1.0 µm and 10–25 µm (for indoor, Fig. 5(a)), which indicates the BC will be from two different kinds of sources in outdoor. Also, finer ranges of BC are found indoors, and the coarser range is found outdoors. So, the finer one observed in the current study might be the vehicular exhaust contributed by roadside vehicles and silent DG sets in industries, and the coarser one may be from the burning of fossil fuel by any other type of industry in the vicinity, which requires further investigation. It is evident from previous research that vehicular exhaust contributes to a fine range (<2.5µm) of particles, where big size BC can be attributed to fossil fuel burning^77^. For indoors, this study observed that the correlation of BC is higher with a wider range of PM than outdoors, which suggests the variation of BC with PM due to the bustle of employees while carrying out industry operations. Comparing the result of the correlation of PM and BC, the observed concentration of PM, the smaller size BC (< 2.5 µm), is about 2/3^rd^ of the total BC concentration.

### Health risk (HR)

The non-carcinogenic HR for the PM_2.5_, PM_10_, BC, and carcinogenic HR (LCR) for BC were quantified for both males and females as per the methodology given in Sect. “Estimation of Health Risk” The HQ estimated over the exposure period (10 days) for PM_2.5_ PM_10,_ and BC was found to be 0.259, 0.375, and 2.25E-04, respectively (considering CPCB and OSHA standards). However, when proposed 8-h annual concentrations indoors (methods as described in Sect. “Air Quality Data and Sampling Methodology” and Eq. 1) were considered, the same risks increased to 0.737 for PM_2.5_ and 0.987 for PM_10_ (Table 3). Further, the estimated results highlighted that the individual risk due to PM and BC were under the safe range (HQ < 1), but the overall noncarcinogenic risk (∑HQ) was found to be 1.72 (> 1), exhibiting a significant health threat. The HQs were also estimated considering WHO reference standards (Annual average), and were found to be 5.89 and 3.95 for PM_2.5_ and PM_10_, respectively, indicating about 4 to 7 times higher risk than HQ considering CPCB standard. This is due to the very stringent permissible annual concentration prescribed by the WHO. Results show that the noncarcinogenic risk may exist for PM, but no significant threat was observed for BC. The LCR for a lower fraction of BC was in the range of 6.17E-05–7.20E-05 when a 10-day average concentration was considered and causing mild carcinogenicity. Concerning carcinogenicity, USEPA considers risk as significant if LCR > 10^–6^, which implies there is a considerable threat for one case over one million population and sufficiently high risk if the risk is greater than one chance in ten thousand persons (1 × 10^–4^). Since the LCR estimated in this study lies in the range of 10^–5^, depicting some probable carcinogenic threat due to BC. This risk may decrease if the monitoring can be done for a longer period.

**Table 3:** Percentage variation between deterministic risk and probabilistic risk due to annual indoor concentration considering RfC by CPCB for PM and RfC by OSHA for BC.

Percentage variation between deterministic risk and probabilistic risk due to annual indoor concentration										
Gender	Age group	Deterministic health risk			Probabilistic health risk			Percentage difference		
		PM_10_	PM_2.5_	*BC	PM_10_	PM_2.5_	*BC	PM_10_	PM_2.5_	*BC
Male	16–21	0.987	0.737	2.25E-04	0.99	0.74	10.4E-05	0.3	0.4	0
	21–30	0.987	0.737	2.25E-4	0.99	0.74	2.25E-4	0.3	0.4	0
	30–40	0.987	0.737	2.25E-4	0.99	0.74	2.25E-4	0.3	0.4	0
	40–50	0.987	0.737	2.25E-4	0.99	0.74	2.25E-4	0.3	0.4	0
	50–60	0.987	0.737	2.25E-4	0.99	0.74	2.26E-4	0.3	0.4	0.4
Female	16–21	0.987	0.737	2.25E-4	0.99	0.74	2.25E-4	0.3	0.4	0
	21–30	0.987	0.737	2.25E-4	0.99	0.74	2.25E-4	0.3	0.4	0
	30–40	0.987	0.737	2.25E-4	0.99	0.74	2.25E-4	0.3	0.4	0
	40–50	0.987	0.737	2.25E-4	0.99	0.74	2.25E-4	0.3	0.4	0
	50–60	0.987	0.737	2.25E-4	0.99	0.74	2.25E-4	0.3	0.4	0

*The two-month daily 8-h average concentration has been considered for estimating the HQ of BC.

As per the deterministic computation, LCR values for BC range from 6.85E-05 to 7.20E-05 and 6.17E-05 to 6.47E-05 for males and females, respectively. Males were found to be at more risk than females, while the males in the age group of 50–60 years and females in the age group of 40–50 years were more vulnerable to carcinogenic risk. The results obtained for LCR due to BC, from both deterministic as well as probabilistic approaches, were compared and presented in Table 4.

**Table 4:** Percentage variation between carcinogenicity risk of BC for a 2-months average indoor concentration using a deterministic and probabilistic approach for risk estimation.

Gender	Age group	Deterministic LCR of BC	Probabilistic LCR of BC	Percentage difference
Males	16–21	6.86E-05	6.93E-05	1.01E-02
	21–30	6.83E-05	6.90E-05	1.02E-02
	30–40	7.19E-05	7.27E-05	1.16E-02
	40–50	7.13E-05	7.22E-05	1.27E-02
	50–60	7.20E-05	7.28E-05	1.12E-02
Females	16–21	6.35E-05	6.42E-05	1.03E-02
	21–30	6.24E-05	6.31E-05	1.05E-02
	30–40	6.17E-05	6.23E-05	0.95E-02
	40–50	6.47E-05	6.54E-05	1.01E-02
	50–60	6.44E-05	6.51E-05	1.13E-02

*The two-month daily 8-h average concentration has been considered for estimating the LCR of BC.

**Fig. 6:**
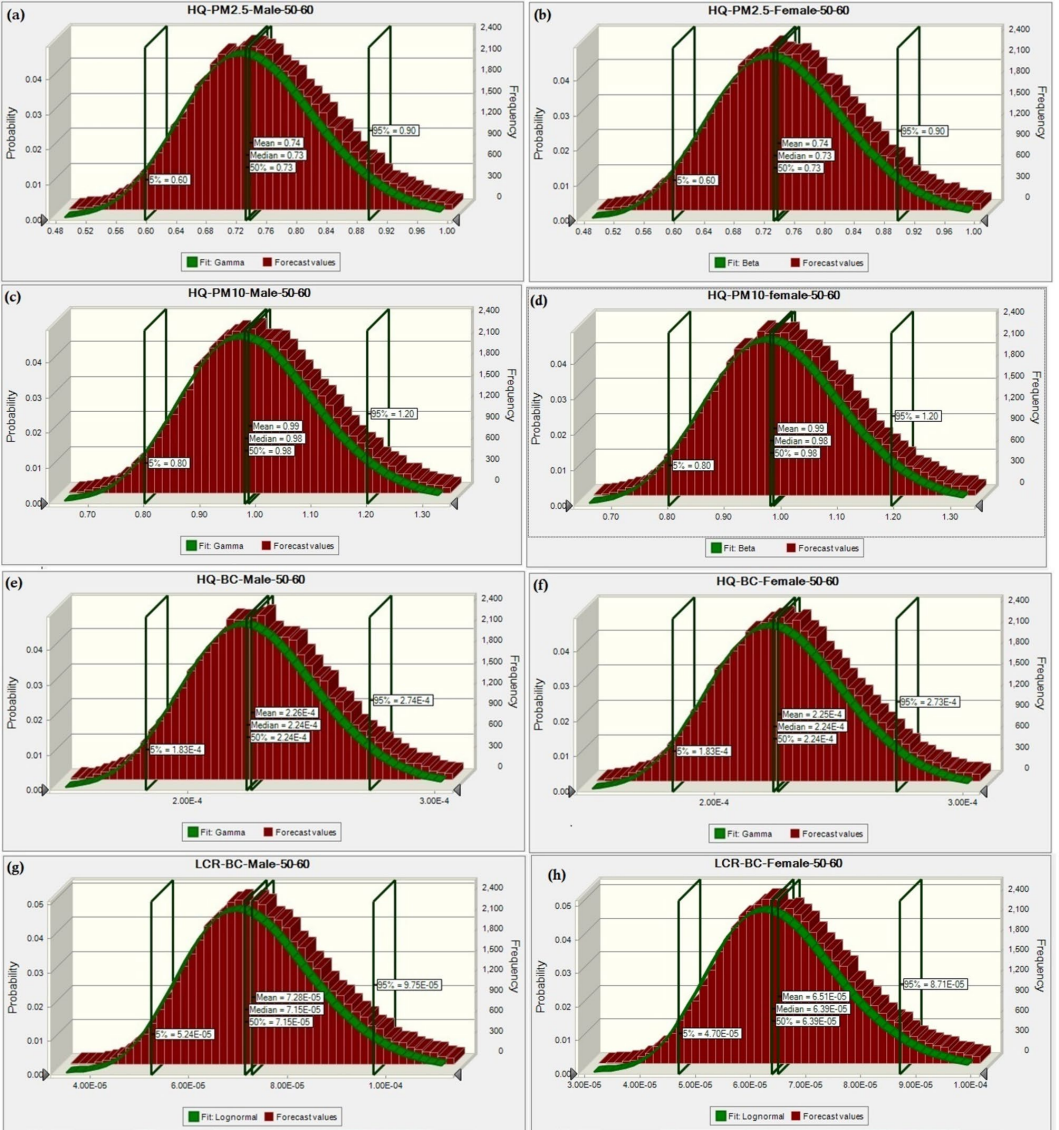
(a-h). Monte Carlo Simulation for probabilistic risk estimation.

Probabilistic computation results (Monte Carlo simulations with 50,000 trials; Fig. 6) also indicated similar patterns of HR values. Moreover, the percentage of variations of HR values from these two computational approaches inferred minor deviations for both males and females in all age groups (Fig. S1-S8). Figure 6 (a to h) shows the probabilistic distribution of PM_2.5_, PM_10,_ and BC for both males and females in the age group of 50–60 years. The maximum variation in the percentage of HR values (1.12E-02)was observed for the age group 40–50 years for males, whereas for females, the variation (1.13E-02) was observed for the age group 50–60.

With the help of both approaches, it was found that a negligible variation of HR (1.13E-02–1.12E-02) exists for PM_10_ and PM_2.5_ for any age or sex. However, for BC, the variation found in the quantification of cancerous risk depicts that males were at higher risk than females. Further, the maximum risk was observed for age categories 50–60 years in males and 40–50 years in females.

**Fig. 7:**
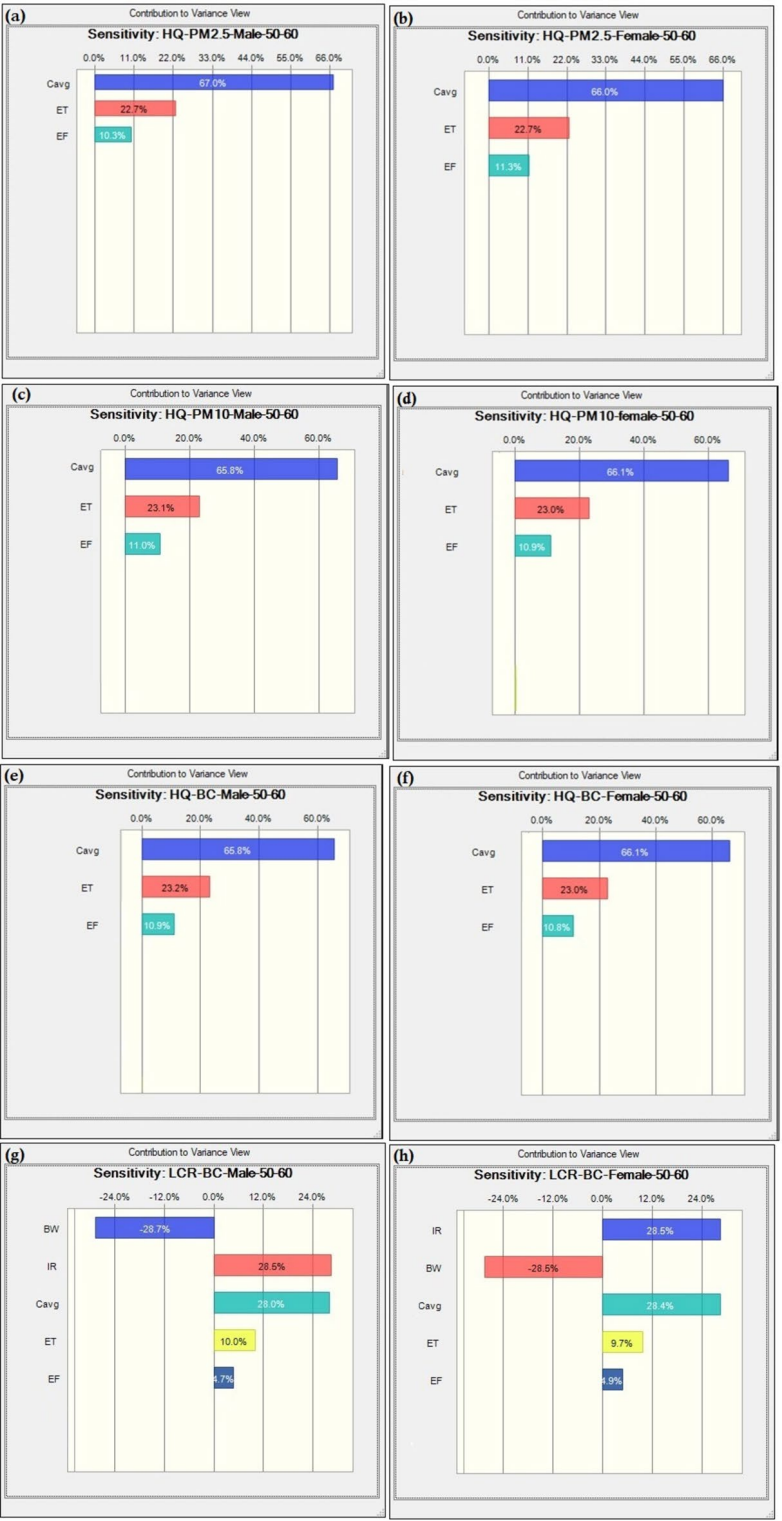
(a-h). Tornado plot of sensitivity analysis.

When the two methods of health risk estimation were compared for each category, the highest uncertainty was seen for the age category 40–50 years in males and the least in the age category of 30–40 years in females for carcinogenic risk due to BC. The minimum risk found for females belonging to the age 30–40 years was 6.17E-05, whereas the maximum risk for males having the age category 16–21 years was estimated to be 6.86E-05. However, to extract each parameter’s importance to the computation of LCR, tornado plots were constructed by performing sensitivity analysis (Fig. 7). From Fig. 7, it is clear that the concentration of PM_2.5_ and PM_10_ played a positive significant role in the HR estimations for male (65.8% to 67.0%) and female (66.0% to 66.1%), followed by ET (23%) and EF (11%). A different trend is observed for the case of BC, where IR, BW, and C_avg_ were the highest significant factors contributing nearly the same weightage (IR, C_avg_;28.5% BW; −28.5%), followed by ET (10%) and EF (4.8%). Notably, body weight is negatively associated with the HR computation for both males (−28.7%) and females (−28.5%), which signifies that increasing human body weight can reduce health risks. The overall results obtained from both deterministic and probabilistic approaches indicate that both approaches are capable of independently estimating human health risk precisely. The results of the sensitivity analysis were found to be different when compared with some other studies. A study carried out by Mohamadi et al.^80^ in Tehran, Iran market, for the health risk assessment of heavy metals in cocoa powder also found pollutant concentration to be the most contributing factor toward health risk, where inhalation rate contributed only about 1–2%, similar to findings of the present study. The concentration of pollutants was found to be the most sensitive factor, contributing approximately 95% in a study carried out in a composite manufacturing plant^81^.

### Spirometry

**Fig. 8:**
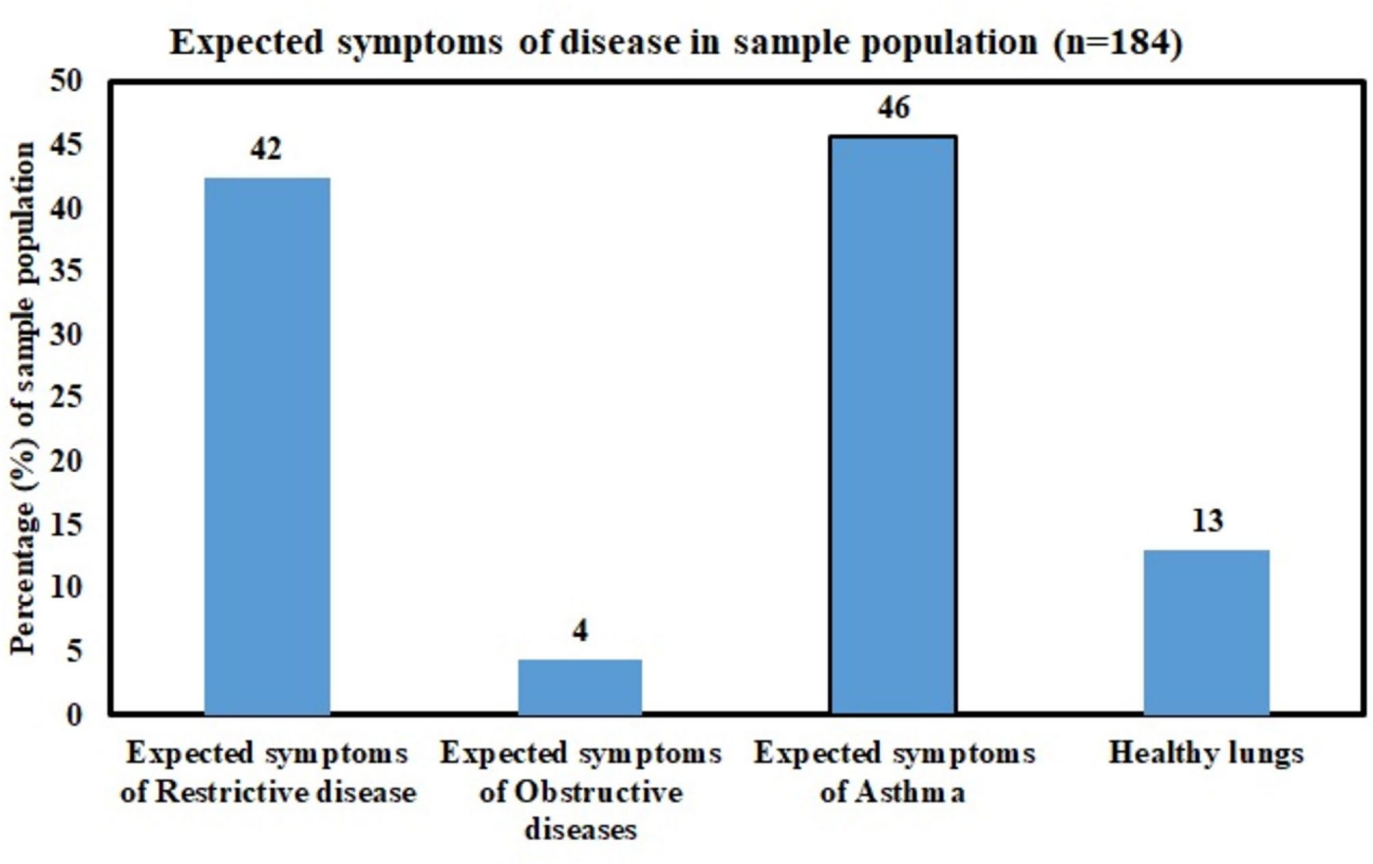
Suspected sample population having symptoms of lung disease.

Spirometry tests were conducted for employees of target industries with the help of a digital spirometer. Three lung function parameters (FVC, FEV_1_/FVC, and PEFR) were recorded for each employee’s LFT, and samples were taken in triplicate. Using the algorithm described in Fig. 2, the pulmonary disorders were dichotomized into three categories, viz., restrictive disease, obstructive disease, and asthma, based on the comparison between predicted and observed values of lung function parameters. The mean FVC, FEV_1_, and PEFR were recorded as 3.43 ± 0.84 (ranges 5.12 to 0.77) litres, 2.98 ± 0.86 (ranges 4.91 to 0.67) liters, and 5.16 ± 2.53 (ranges 12.7 to 0.88) liters/sec respectively, among the sample population (n = 184). Based on the quantitative analysis, 13.04% of the sample population was found to have healthy lungs, whereas the rest are suspected to be affected by lung diseases. Also, the estimation shows that 42.39%, 4.34%, and 53.26% of the total sample population (n = 184) might be suffering from restrictive disease, obstructive disease, and asthma, respectively, as depicted in Fig. 8 and Table 5. Expected cases of asthma were most prevalent, followed by expected cases of restrictive disease and obstructive disease. Since the PM and BC concentrations were found to be more than their permissible limits, their excess concentration may also be considered a contributing factor toward lung diseases. Many studies found elevated PM concentrations attributed to lung impairments. A study carried out by Liu et al. and Sharma et al.^82,83^ in Kanpur city of India found an increased number of pulmonary hospital visits with an increase in PM concentration in the year 2006. Another study of industries in Delhi found a significant positive correlation between declines in the respiratory health of industrial workers with elevated PM_2.5_ levels^84^. Survey-based research conducted in a brick kiln in Jalalpur Jattan, Pakistan, revealed the decay of lung function associated with an increase in PM levels^85^. Similar results were also reported by some researchers in Chuncheon City, Korea^86^, the northeast region of China^87^, and Benin City, Nigeria^59^. On the other hand, PM is not the only factor responsible for lung diseases; it could be attributed to many other factors such as smoking, fuel usage at home, exercise, etc^53,88,89^.

**Table 5:** Demographic and lung function parameters variation.

Total no. of cases (184), No. of workers (100), No. of staff members (84), Cases of alcohol (78), Cases of smoking (92)			
	Minimum	Average	Maximum
Height (cm)	145	163 ± 7	180
Weight (Kg)	40	61 ± 15	105
Age (years)	18	31 ± 11	65
Industrial working hours	8	8.5 ± 1.5	12
Working time in industries(years)	0.2	5.4 ± 5.1	23
FVC (liters)	0.77	3.43 ± 0.83	5.12
FEV1 (liters)	0.67	2.98 ± 0.86	4.91
PEFR (liters/second)	0.88	5.16 ± 2.53	12.7

**Fig. 9:**
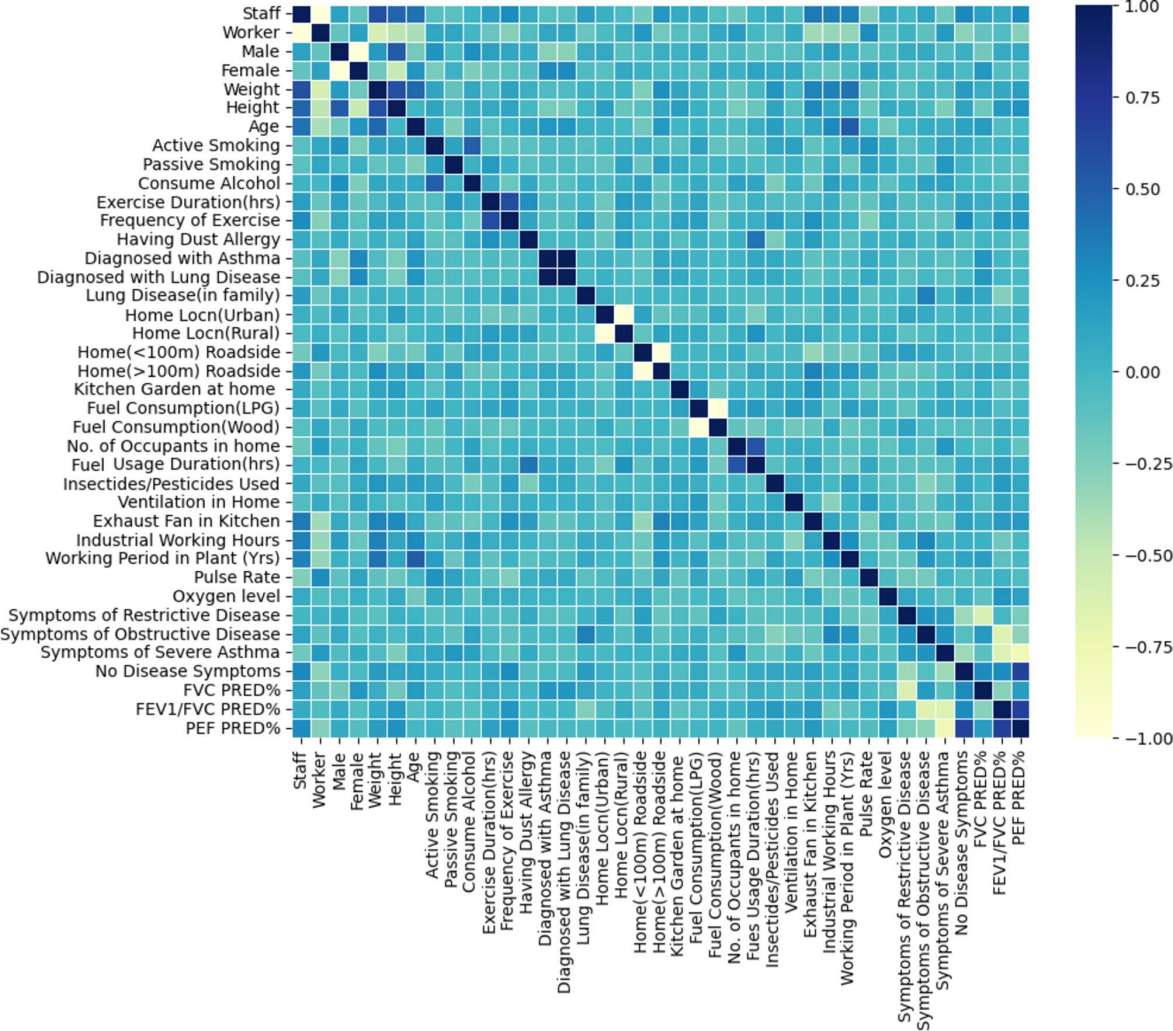
Correlation matrix of responses and lung disorder.

Most of the researchers intend to find the impact of air pollutants on lung health or try to correlate their lifestyle exposure conditions to lung parameters, but few researchers have tried to incorporate most of them. This study considered a holistic approach, including monitoring of pollutants, an assessment of lung function parameters, and a questionnaire survey. To assess the contribution of factors other than air pollutants, towards lung condition, the responses to the questionnaire-based survey were attached with spirometry results for individuals, and each factor was correlated with the symptom of lung disease using Pearson coefficient of correlation and results are represented in Fig. 9. However, a bivariate correlation considering one tail test was also performed to catch the statistically significant factors. We found a positive correlation between asthma symptoms for workers (r = 0.18, p < 0.05) but not with staff members. It was also found that the decrement of lung capabilities was due to increased PM and BC concentrations, as workers were openly exposed to the working area while staff members were allotted closed cabins where such high concentrations were not available. PEFR was positively correlated with height (r = 0.237, p < 0.05), but no statistically significant correlation was found with lung diseases in our study. From the control group, 26.1% were active smokers, out of which 83.14% were found to have symptoms of asthma, which may indicate that smoking is a responsible factor for a pulmonary disorder. Similar results showing smoking to be an attributable factor for lung degradation were also drawn by other researchers^90–93^. Vanesa, in his study, found that smokers display an increased prevalence and incidence of asthma, but a causal association cannot be claimed using existing evidence. The same study also considers the positive linkage of passive smoking with the incidence of asthma. Physical exercise shows a good correlation with healthy lungs, concluding that frequent physical exercise can keep lungs healthy. Also, statistical analyses decline the impact of ‘alcohol consumption, home location in rural or urban, home location proximity to the road, the kitchen garden at home, use of insecticides or pesticides at home and exhaust fan in the kitchen’ on the lung health condition. Stapleton et al.^94^ investigated that using LPG or biomass in kitchens for cooking purposes results in high concentrations of fine PM and consequently results in the degradation of lungs, while the current study does not find such inferences. In a study carried out in the HBA industrial area of northern Israel, asthma is highly associated with industrial pollution^31^.

**Fig. 10:**
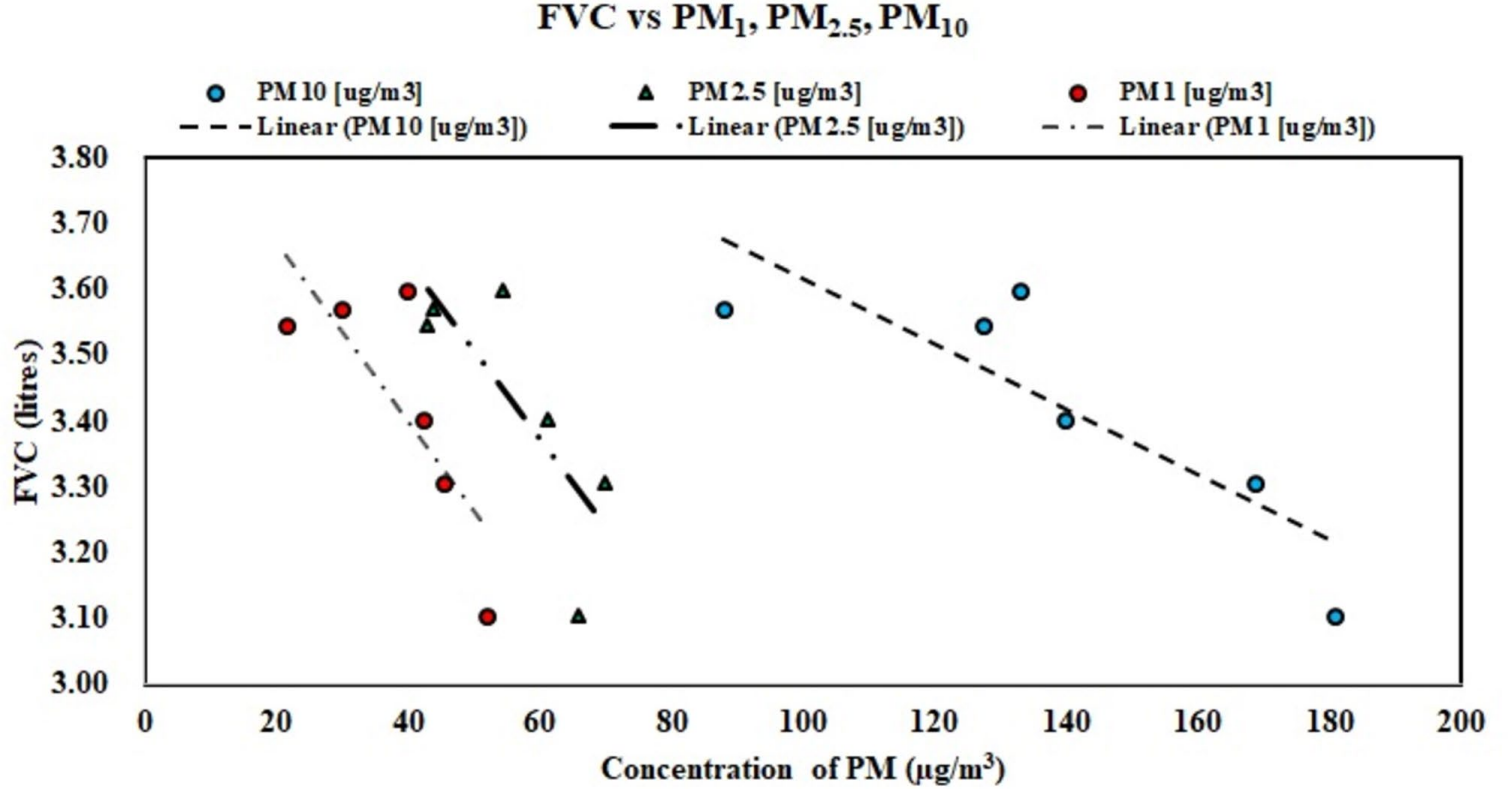
Trend of FVC to change in PM concentrations.

Comparing the FVC found from the employees and workers of five industries with the concentration of PM (Fig. 10), the ultrafine particles, along with PM_2.5_, have a greater impact on FVC. This is corroborated by Li et al.^95^. Further considering the impact of similar concentrations (say for 50 µg/m3) of PM_1_ and PM_2.5_ on FVC, it is found that PM_2.5_ has less effect than PM_1,_ which can further be clarified by proper investigation through characterizations of PM. Similar findings (lower FVC with an increase in PM_1_ concentration) are reported by other researchers^96,97^ adding to the adverse impact on lung function.

### Limitations of this study

There is insufficient published data on inhalation rate and body weight for various age groups in the Indian context, and the standard average body weight and inhalation data, as provided by USEPA 2011, were adopted to estimate the health risk. Additionally, the sample size (148) used for this study may not be enough to generalize the body weight and inhalation rate for various age groups in the Indian scenario. Further, the data presented were taken for 10 days, spreading over two months in the post-monsoon season (September and October) of 2022, as there are constraints on access to obtain daily data by entering the restricted area of those industries for a long time. This study is cross-sectional in design; hence, to confirm the association of health risks due to the observed pollutants, a thorough study for a longer time is warranted.

## Conclusion

This study suggests that the indoor and outdoor environment in the study area encompassed high concentrations of PM and BC, where PM concentrations exceeded the permissible limits set by CPCB and WHO. Further, the indoor concentrations were found to be higher than outdoor concentrations, posing more health threats, and ambient pollutants have a major contribution to indoor concentrations. Moreover, the reported correlation of BC with particle ranges of 10 to 32 microns needs to be investigated through a characterization study of PM followed by a source apportionment study as future research. The risk assessment demonstrated that the employees of industries were at a considerable non-carcinogenic health risk due to PM_10_ and PM_2.5_ but can not be denied for carcinogenic risk due to BC having variation with age and sex. Health risk analysis for PM_10_, PM_2.5,_ and BC using both deterministic and probabilistic approaches provided similar results with very marginal variations. Therefore, both approaches can be utilized individually for the computation of human health risk. The spirometry results elucidated the bad condition of the worker’s lungs, which could be attributed to elevated PM and BC concentrations, but it can not be concluded without clinical evidence because lung diseases are attributed to the weightage of multiple factors. The outcomes of this study will assist in the call for the public’s and policymakers’ attention to increased levels of PM and BC and control of pulmonary disorders in employees in the study area. One of the solutions may be creating a green belt surrounding industries which are situated close to roadsides and opting for sufficient ventilation inside the industries. It is also recommended that a technical audit be conducted quarterly, and during the audit period, indoor pollutants should be monitored for at least a week (including weekends and weekdays) in each season to enable the proper assessment of health risks.

## Supplementary Information

Supplementary Information.

## Data Availability

The datasets used and/or analysed during the current study available from the corresponding author on reasonable request.

## References

[1] Geneva: World Health Organization. WHO global air quality guidelines. *Part. matter (PM2.5 PM10), ozone, nitrogen dioxide, sulfur dioxide carbon monoxide* 1–360 (2021). 34662007

[2] Nordeide Kuiper, I. et al. Lifelong exposure to air pollution and greenness in relation to asthma, rhinitis and lung function in adulthood. *Environ. Int.* **146**, 106219 (2021). 33126061 10.1016/j.envint.2020.106219

[3] Anwar, M. N. et al. Emerging challenges of air pollution and particulate matter in China, India, and Pakistan and mitigating solutions. *J. Hazard. Mater.* **416**, 125851 (2021). 34492802 10.1016/j.jhazmat.2021.125851

[4] Nardocci, A. C., Nogueira, T., de Almeida Piai, K., Cavendish, T. A. & Kumar, P. Indoor environment exposure and children’s health. *Curr. Opin. Environ. Sci. Heal.* **32**, 100449 (2023).

[5] Ali, M. U. et al. Health impacts of indoor air pollution from household solid fuel on children and women. *J. Hazard. Mater.* **416**, 126127 (2021). 34492921 10.1016/j.jhazmat.2021.126127

[6] Baldelli, A. Evaluation of a low-cost multi-channel monitor for indoor air quality through a novel, low-cost, and reproducible platform. *Meas. Sensors* **17**, 100059 (2021).

[7] Singh, D. et al. Statistical modeling of O3, NOx, CO, PM2.5, VOCs and noise levels in commercial complex and associated health risk assessment in an academic institution. *Sci. Total Environ.* **572**, 586–594 (2016). 27575044 10.1016/j.scitotenv.2016.08.086

[8] González-Martín, J., Kraakman, N. J. R., Pérez, C., Lebrero, R. & Muñoz, R. A state–of–the-art review on indoor air pollution and strategies for indoor air pollution control. *Chemosphere* **262**, 128376 (2021). 33182138 10.1016/j.chemosphere.2020.128376

[9] Cohen, A. J. et al. Estimates and 25-year trends of the global burden of disease attributable to ambient air pollution: An analysis of data from the global burden of diseases study 2015. *Lancet* **389**, 1907–1918 (2017). 28408086 10.1016/S0140-6736(17)30505-6 PMC5439030

[10] Singh, A. A. et al. Assessment of ozone toxicity among 14 Indian wheat cultivars under field conditions: growth and productivity. *Environ. Monit. Assess.*10.1007/s10661-018-6563-0 (2018). 29502252 10.1007/s10661-018-6563-0

[11] Kirrane, E. F. et al. A systematic review of cardiovascular responses associated with ambient black carbon and fine particulate matter. *Environ. Int.* **127**, 305–316 (2019). 30953813 10.1016/j.envint.2019.02.027 PMC8517909

[12] Kampa, M. & Castanas, E. Human health effects of air pollution. *Environ. Pollut.* **151**, 362–367 (2008). 17646040 10.1016/j.envpol.2007.06.012

[13] Valavanidis, A., Fiotakis, K. & Vlachogianni, T. Airborne particulate matter and human health: Toxicological assessment and importance of size and composition of particles for oxidative damage and carcinogenic mechanisms. *J. Environ. Sci. Heal. - Part C Environ*. *Carcinog. Ecotoxicol. Rev.* **26**, 339–362 (2008). 10.1080/10590500802494538 19034792

[14] Janssen, N. A. H. et al. Black carbon as an additional indicator of the adverse health effects of airborne particles compared with pm10 and pm2.5. *Environ. Health Perspect.* **119**, 1691–1699 (2011). 21810552 10.1289/ehp.1003369 PMC3261976

[15] Kwon, H. S., Ryu, M. H. & Carlsten, C. Ultrafine particles: unique physicochemical properties relevant to health and disease. *Exp. Mol. Med.* **52**, 318–328 (2020). 32203103 10.1038/s12276-020-0405-1 PMC7156720

[16] Paunescu, A. C. et al. Associations of black carbon with lung function and airway inflammation in schoolchildren. *Environ. Int.* **131**, 104984 (2019). 31301585 10.1016/j.envint.2019.104984

[17] Kahnert, M. & Kanngießer, F. Modelling optical properties of atmospheric black carbon aerosols. *J. Quant. Spectrosc. Radiat. Transf.* **244**, 106849 (2020).

[18] CCA. Black carbon | Climate & Clean Air Coalition. *Climate and Clean Air Coalition* (2022).

[19] Yang, J. et al. Long-term exposure to black carbon and mortality: A 28-year follow-up of the GAZEL cohort. *Environ. Int.* **157**, 106805 (2021). 34375941 10.1016/j.envint.2021.106805

[20] Vu, T. V. et al. Assessing the contributions of outdoor and indoor sources to air quality in London homes of the SCAMP cohort. *Build. Environ.* **222**, 109359 (2022).

[21] Mansor, A. A. et al. Indoor air quality and sick building syndrome symptoms in administrative office at public university. *Dialogues Heal.* **4**, 100178 (2024). 10.1016/j.dialog.2024.100178 PMC11043824 38665133

[22] Ambade, B., Sankar, T. K., Panicker, A. S., Gautam, A. S. & Gautam, S. Characterization, seasonal variation, source apportionment and health risk assessment of black carbon over an urban region of East India. *Urban Clim.* **38**, 100896 (2021).

[23] Wang, L. et al. Low-dose exposure to black carbon significantly increase lung injury of cadmium by promoting cellular apoptosis. *Ecotoxicol. Environ. Saf.* **224**, 112703 (2021). 34479021 10.1016/j.ecoenv.2021.112703

[24] Di Ianni, E., Jacobsen, N. R., Vogel, U. B. & Møller, P. Systematic review on primary and secondary genotoxicity of carbon black nanoparticles in mammalian cells and animals. *Mutat. Res. - Rev. Mutat. Res.* **790**, 108441 (2022). 36007825 10.1016/j.mrrev.2022.108441

[25] Ali, M. U. et al. Emission sources and full spectrum of health impacts of black carbon associated polycyclic aromatic hydrocarbons (PAHs) in urban environment: A review. *Crit. Rev. Environ. Sci. Technol.* **51**, 857–896 (2021).

[26] Lepistö, T. et al. Connection between lung deposited surface area (LDSA) and black carbon (BC) concentrations in road traffic and harbour environments. *Atmos. Environ.* **272**, 118931 (2022).

[27] Collaro, A. J. et al. Associations between lung function and future cardiovascular morbidity and overall mortality in a predominantly First Nations population: A cohort study. *Lancet Reg. Heal. - West. Pacific* **13**, 100188 (2021). 10.1016/j.lanwpc.2021.100188 PMC8403916 34527981

[28] Ferreira Nunes, M., Plácido da Silva, H., Raposo, L. & Rodrigues, F. Design and Evaluation of a Novel Venturi-Based Spirometer for Home Respiratory Monitoring. *Sensors* **24**, 5622 (2024). 39275533 10.3390/s24175622 PMC11398045

[29] Hoesterey, D. et al. Spirometric indices of early airflow impairment in individuals at risk of developing COPD: Spirometry beyond FEV1/FVC. *Respir. Med.* **156**, 58–68 (2019). 31437649 10.1016/j.rmed.2019.08.004 PMC6768077

[30] Naik, M., Amin, A., Gani, M., Bhat, T. A. & Wani, A. A. Effect of automobile exhaust on pulmonary function tests among traffic police personnel in Kashmir valley. *Lung India* **39**, 116 (2022). 35259793 10.4103/lungindia.lungindia_323_21 PMC9053919

[31] Raz, R. et al. Associations between exposure to industrial air pollution and prevalence of asthma and atopic diseases in haifa bay area. *Atmosphere (Basel).* **12**, 1–23 (2021).

[32] Kanawade, V. P. et al. What caused severe air pollution episode of November 2016 in New Delhi?. *Atmos. Environ.* **222**, 117125 (2020).

[33] Manojkumar, N. & Srimuruganandam, B. Health effects of particulate matter in major Indian cities. *Int. J. Environ. Health Res.* **31**, 258–270 (2021). 31392891 10.1080/09603123.2019.1651257

[34] Ayejoto, D. A., Agbasi, J. C., Nwazelibe, V. E., Egbueri, J. C. & Alao, J. O. Understanding the connections between climate change, air pollution, and human health in Africa: Insights from a literature review. *J. Environ. Sci. Heal. Part C Toxicol. Carcinog.* **41**, 77–120 (2023). 10.1080/26896583.2023.2267332 37880976

[35] Haynes. Chapter 9 Chapter 9. *Cycle* **1897**, 44–45 (2021).

[36] Hayes, G., Dowd, K. P., MacDonncha, C. & Donnelly, A. E. Tracking of Physical activity and sedentary behavior from adolescence to young adulthood: A systematic literature review. *J. Adolesc. Heal.* **65**, 446–454 (2019). 10.1016/j.jadohealth.2019.03.013 31248803

[37] U.S. Environmental Protection Agency. Exposure factors Handbook : 2011 Edition National Center for Environmental Assessment Office of Research and Development U. S. Environmental Protection Agency Washington , DC 20460 Exposure Factors Handbook. *Expo. Factors Handb. 2011 USEPA* (2011).

[38] U.S. Environmental Protection Agency (EPA). (2011). Exposure Factors Handbook: 2011 Edition. National Center for Environmental Assessment, Washington, DC: EPA/600/R-09/052F

[39] Deng, W. J., Zheng, H. L., Tsui, A. K. Y. & Chen, X. W. Measurement and health risk assessment of PM2.5, flame retardants, carbonyls and black carbon in indoor and outdoor air in kindergartens in Hong Kong. *Environ. Int.* **96**, 65–74 (2016). 27608428 10.1016/j.envint.2016.08.013

[40] Li, G., Wu, S., Wang, L. & Akoh, C. C. Concentration, dietary exposure and health risk estimation of polycyclic aromatic hydrocarbons (PAHs) in youtiao, a Chinese traditional fried food. *Food Control* **59**, 328–336 (2016).

[41] Bakhtiari, A. R., Zakaria, M. P., Yaziz, M. I., Lajis, M. N. H. L. & Bi, X. Environment Asia. *Environ. Asia* **7**, 104–111 (2014).

[42] Lequy, E. et al. Contribution of long-term exposure to outdoor black carbon to the carcinogenicity of air pollution: Evidence regarding risk of cancer in the gazel cohort. *Environ. Health Perspect.*10.1289/EHP8719 (2021). 33759553 10.1289/EHP8719 PMC7989243

[43] EPA. Science Policy Council Handbook: Risk characterization. 31 (2000).

[44] Khan, M. D. H., Sarkar, M. S., Haque, S. S. & Hossain, M. A. Health risk assessment of black carbon emission from fossil fuel. *J. Eng. Sci.* **12**, 23–28 (2021).

[45] US EPA. Regional Screening Levels (RSLs) - Generic Tables (November 2015). *United States Environmental Protection Agency* (2015).

[46] Lonati, G. & Zanoni, F. Probabilistic health risk assessment of carcinogenic emissions from a MSW gasification plant. *Environ. Int.* **44**, 80–91 (2012). 22364891 10.1016/j.envint.2012.01.013

[47] Zhang, T. *et al.* Band selection in sentinel-2 satellite for agriculture applications. *ICAC 2017 - 2017 23rd IEEE Int. Conf. Autom. Comput. Addressing Glob. Challenges through Autom. Comput.*10.23919/iconac.2017.8081990 (2017).

[48] Kaur, L., Rishi, M. S. & Siddiqui, A. U. Deterministic and probabilistic health risk assessment techniques to evaluate non-carcinogenic human health risk (NHHR) due to fluoride and nitrate in groundwater of Panipat, Haryana. *India. Environ. Pollut.* **259**, 113711 (2020). 31891909 10.1016/j.envpol.2019.113711

[49] Pasupuleti, S. et al. Groundwater characterization and non-carcinogenic and carcinogenic health risk assessment of nitrate exposure in the Mahanadi River Basin of India. *J. Environ. Manage.* **319**, 115746 (2022). 35982575 10.1016/j.jenvman.2022.115746

[50] Roca, J. et al. References values for forced spirometry. *Eur. Respir. J.* **11**, 1354–1362 (1998). 9657579 10.1183/09031936.98.11061354

[51] Harvey, B. G. et al. Risk of COPD with obstruction in active smokers with normal spirometry and reduced diffusion capacity. *Eur. Respir. J.* **46**, 1589–1597 (2015). 26541521 10.1183/13993003.02377-2014 PMC4752006

[52] McCracken, J. L., Veeranki, S. P., Ameredes, B. T. & Calhoun, W. J. Diagnosis and management of asthma in adults: A review. *JAMA* **318**, 279–290 (2017). 28719697 10.1001/jama.2017.8372

[53] Siddique, S., Banerjee, M., Ray, M. R. & Lahiri, T. Air pollution and its impact on lung function of children in Delhi, the capital city of India. *Water. Air. Soil Pollut.* **212**, 89–100 (2010).

[54] NIH. Guidelines for the Diagnosis and Management of Asthma 2007 (EPR-3) | NHLBI, NIH. (2007).

[55] CPCB. *Guidelines for the Measurement of Ambient Air Pollutants Volume-I NAAQMS/36/2012–13*. (2012).

[56] WHO. *Evolution of WHO air quality guidelines: past, present and future*. *Copenhagen: WHO Regional Office for Europe* (2017).

[57] Heydari, G. *et al.* Levels and health risk assessments of particulate matters (PM 2.5 and PM 10 ) in indoor/outdoor air of waterpipe cafés in Tehran, Iran. *Environ. Sci. Pollut. Res.* **26**, 7205–7215 (2019). 10.1007/s11356-019-04202-5 30656582

[58] Habil, M. & Taneja, A. Children’s exposure to indoor particulate matter in naturally ventilated schools in India. *Indoor Built Environ.* **20**, 430–448 (2011).

[59] Eghomwanre, A. F., Oguntoke, O. & Taiwo, A. M. Levels of indoor particulate matter and association with asthma in children in Benin City, Nigeria. *Environ. Monit. Assess.*10.1007/s10661-022-10135-3 (2022). 35648237 10.1007/s10661-022-10135-3

[60] Yang, M. et al. Concentration, chemical composition and toxicological responses of the ultrafine fraction of urban air particles in PM1. *Environ. Int.* **170**, 107661 (2022).

[61] Li, Y. et al. Emergency department visits in children associated with exposure to ambient PM1 within several hours. *Int. J. Environ. Res. Public Health* **20**, 1–11 (2023). 10.3390/ijerph20064910 PMC10049417 36981834

[62] Chithra, V. S. & Shiva Nagendra, S. M. Indoor air quality investigations in a naturally ventilated school building located close to an urban roadway in Chennai. *India. Build. Environ.* **54**, 159–167 (2012).

[63] Dorizas, P. V., Assimakopoulos, M. N., Helmis, C. & Santamouris, M. An integrated evaluation study of the ventilation rate, the exposure and the indoor air quality in naturally ventilated classrooms in the Mediterranean region during spring. *Sci. Total Environ.* **502**, 557–570 (2015). 25300020 10.1016/j.scitotenv.2014.09.060

[64] Qadr, S. R. Impact assessment of particulate matter (PM 10) by Cement Industry: A case study in khrew & khanmoh (J&K). *India.*10.21203/RS.3.RS-2611835/V1 (2023).

[65] Singh, L. P., Bhardwaj, A. & Deepak, K. K. Respirable suspended particulate matter (RSPM) and respiratory symptom among casting industry workers: An exploratory study in Northern India. *Int. J. Adv. Eng. Technol.* **2**, 251–259 (2011).

[66] Semple, S., Green, D. A., McAlpine, G., Cowie, H. & Seaton, A. Exposure to particulate matter on an Indian stone-crushing site. *Occup. Environ. Med.* **65**, 300–305 (2008). 17681995 10.1136/oem.2007.032805

[67] Sui, X., Tian, Z., Liu, H., Chen, H. & Wang, D. Field measurements on indoor air quality of a residential building in Xi’an under different ventilation modes in winter. *J. Build. Eng.* **42**, 103040 (2021).

[68] Ravindra, K. Emission of black carbon from rural households kitchens and assessment of lifetime excess cancer risk in villages of North India. *Environ. Int.* **122**, 201–212 (2019). 30522824 10.1016/j.envint.2018.11.008

[69] Viidanoja, J. et al. Organic and black carbon in PM2.5 and PM10: 1 Year of data from an urban site in Helsinki. *Finland. Atmos. Environ.* **36**, 3183–3193 (2002).

[70] Sarkar, C., Roy, A., Chatterjee, A., Ghosh, S. K. & Raha, S. Factors controlling the long-term (2009–2015) trend of PM2.5 and black carbon aerosols at eastern Himalaya. *India. Sci. Total Environ.* **656**, 280–296 (2019). 30513422 10.1016/j.scitotenv.2018.11.367

[71] Liang, F. et al. Associations of PM2.5 and black carbon with hospital emergency room visits during heavy haze events: A case study in Beijing, China. *Int. J. Environ. Res. Public Health* **14**, 725 (2017). 28678202 10.3390/ijerph14070725 PMC5551163

[72] Bell, M. L., Zanobetti, A. & Dominici, F. Evidence on vulnerability and susceptibility to health risks associated with short-term exposure to particulate matter: A systematic review and meta-analysis. *Am. J. Epidemiol.* **178**, 865–876 (2013). 23887042 10.1093/aje/kwt090 PMC3775545

[73] Wang, X. et al. Associations between fine particle, coarse particle, black carbon and hospital visits in a Chinese city. *Sci. Total Environ.* **458–460**, 1–6 (2013). 23639905 10.1016/j.scitotenv.2013.04.008

[74] Tane, E. G., Amorós-Pérez, A., Martínez-Gómez, L., Román-Martínez, M. C. & Lillo-Ródenas, M. A. Review and comparative analysis of the particulate matter generated in conventional cigarettes and heated tobacco products - mainstream and environmental emissions. *Environ. Adv.* **16**, 100552 (2024).

[75] Park, G. et al. Characterizing black carbon emissions from gasoline, LPG, and diesel vehicles via transient chassis-dynamometer tests. *Appl. Sci.* **10**, 1–15 (2020).

[76] Paladiychuk, Y., Telyatnuk, I. & Buzdygan, M. Study of smoke of exhaust gasses of internal combustion engines operating on diesel fuel with biople elements. *Eng. Energy, Transp. AIC*10.37128/2520-6168-2022-2-8 (2022).

[77] Tiwari, S., Pipal, A. S., Srivastava, A. K., Bisht, D. S. & Pandithurai, G. Determination of wood burning and fossil fuel contribution of black carbon at Delhi, India using aerosol light absorption technique. *Environ. Sci. Pollut. Res.* **22**, 2846–2855 (2015). 10.1007/s11356-014-3531-2 25217282

[78] Gramsch, E. et al. Particle size distribution and its relationship to black carbon in two urban and one rural site in Santiago de Chile. *J. Air Waste Manag. Assoc.* **64**, 785–796 (2014). 25122952 10.1080/10962247.2014.890141

[79] Raparthi, N. & Phuleria, H. C. Real-world vehicular emissions in the Indian megacity: Carbonaceous, metal and morphological characterization, and the emission factors. *Urban Clim.* **39**, 1–16 (2021).

[80] Mohamadi, S. et al. Probabilistic health risk assessment of heavy metals (Cd, Pb, and As) in Cocoa powder (Theobroma cacao) in Tehran, Iran market. *Int. J. Environ. Health Res.* **34**, 257–272 (2024). 36395480 10.1080/09603123.2022.2146070

[81] Khoshakhlagh, A. H., Askari Majdabadi, M., Yazdanirad, S. & Carlsen, L. Health risk assessment of exposure to benzene toluene ethylbenzene and xylene (BTEX) in a composite manufacturing plant: Monte-Carlo simulations. *Hum. Ecol. Risk Assess.* **0**, 1–15 (2023).

[82] Liu, H. Y. et al. Respiratory disease in relation to outdoor air pollution in Kanpur. *India. Arch. Environ. Occup. Heal.* **68**, 204–217 (2013). 10.1080/19338244.2012.701246 PMC3678152 23697693

[83] Sharma, M. et al. Effects of particulate air pollution on the respiratory health of subjects who live in three areas in Kanpur India. *Arch. Environ. Health: Int. J.* **59**, 348–358 (2010). 10.3200/AEOH.59.7.348-358 16241039

[84] Parveen, N. et al. Industries in Delhi: Air pollution versus respiratory morbidities. *Process Saf. Environ. Prot.* **152**, 495–512 (2021).

[85] Raza, A. et al. Particulate matter associated lung function decline in brick kiln workers of Jalalpur Jattan. *Pakistan. Pak. J. Zool.* **46**, 237–243 (2014).

[86] Jo, Y. S., Lim, M. N., Han, Y. J. & Kim, W. J. Epidemiological study of PM2.5and risk of COPD-related hospital visits in association with particle constituents in Chuncheon, Korea. *Int. J. COPD* **13**, 299–307 (2018). 10.2147/COPD.S149469 PMC5769598 29391787

[87] Yang, M. et al. Is PM1 similar to PM2.5? A new insight into the association of PM1 and PM2.5 with children’s lung function. *Environ. Int.* **145**, 106092 (2020). 32916413 10.1016/j.envint.2020.106092

[88] Sharma, A. K., Baliyan, P. & Kumar, P. Air pollution and public health: The challenges for Delhi. *India. Rev. Environ. Health* **33**, 77–86 (2018). 29267177 10.1515/reveh-2017-0032

[89] Mishra, V. Effect of indoor air pollution from biomass combustion on prevalence of asthma in the elderly. *Environ. Health Perspect.* **111**, 71–78 (2003). 12515681 10.1289/ehp.5559 PMC1241308

[90] Bellou, V., Gogali, A. & Kostikas, K. Asthma and Tobacco Smoking. *J. Pers. Med.* **12**, 1231 (2022). 36013180 10.3390/jpm12081231 PMC9409665

[91] Sangani, R. G. et al. Interstitial lung abnormalities and interstitial lung diseases associated with cigarette smoking in a rural cohort undergoing surgical resection. *BMC Pulm. Med.* **22**, 1–14 (2022). 35488260 10.1186/s12890-022-01961-9 PMC9055776

[92] Heloma, A., Nurminen, M., Reijula, K. & Rantanen, J. Smoking prevalence, smoking-related lung diseases, and national tobacco control legislation. *Chest* **126**, 1825–1831 (2004). 15596680 10.1378/chest.126.6.1825

[93] Tashkin, D. P. & Roth, M. D. Pulmonary effects of inhaled cannabis smoke. *Am. J. Drug Alcohol Abuse* **45**, 596–609 (2019). 31298945 10.1080/00952990.2019.1627366

[94] Stapleton, E. M. et al. Lung function of primary cooks using LPG or biomass and the effect of particulate matter on airway epithelial barrier integrity. *Environ. Res.* **189**, 109888 (2020). 32979995 10.1016/j.envres.2020.109888 PMC7525042

[95] Li, Q. Q., Guo, Y. T., Yang, J. Y. & Liang, C. S. Review on main sources and impacts of urban ultrafine particles: Traffic emissions, nucleation, and climate modulation. *Atmos. Environ. X* **19**, 100221 (2023).

[96] Wu, H. et al. Association between short-term exposure to ambient PM1 and PM2.5 and forced vital capacity in Chinese children and adolescents. *Environ. Sci. Pollut. Res.* **29**, 71665–71675 (2022). 10.1007/s11356-022-20842-6 35604593

[97] Xing, X. et al. Interactions between ambient air pollution and obesity on lung function in children: The Seven Northeastern Chinese Cities (SNEC) Study. *Sci. Total Environ.* **699**, 134397 (2020). 31677469 10.1016/j.scitotenv.2019.134397

