# Supplementary material for: Expected health risk out of black carbon and particulate matter in the indoor environment of an industrial cluster of chandigarh in India
Source: Sci Rep. 2025 Jul 2;15:23177. doi: 10.1038/s41598-025-01606-x (PMC12222698; doi:10.1038/s41598-025-01606-x)
Supplement: Supplementary file 1 — Supplementary Information. [file 41598_2025_1606_MOESM1_ESM.docx]

**Table S1**

RfC of PM set by different regulatory bodies in µg/m^3^

| Pollutants | NAAQS- CPCB (Daily average) | NAAQS- CPCB (Anual average) | WHO(2021) (Daily average) | WHO(2021) (Anual average) | OSHA-NIOSH (8-hour average) |
| --- | --- | --- | --- | --- | --- |
| **PM_2.5_** | 60 | 40 | 15 | 5 | - |
| **PM_10_** | 100 | 60 | 45 | 15 | - |
| **BC** | - | - | - | - | 3500 |
